# Supplementary material for: High content screening and proteomic analysis identify a kinase inhibitor that rescues pathological phenotypes in a patient-derived model of Parkinson’s disease
Source: NPJ Parkinsons Dis. 2022 Feb 11;8:15. doi: 10.1038/s41531-022-00278-y (PMC8837749; doi:10.1038/s41531-022-00278-y)
Supplement: Supplementary file 1 — Supplementary information [file 41531_2022_278_MOESM1_ESM.pdf]

a

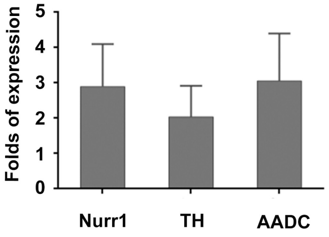

b

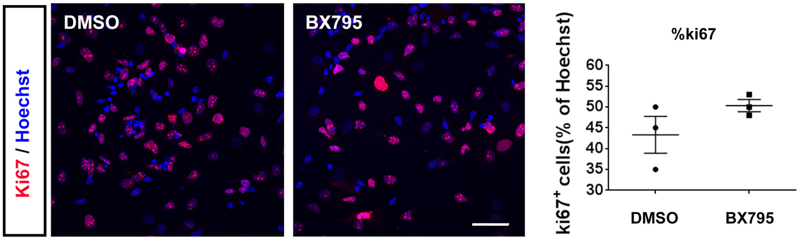

c

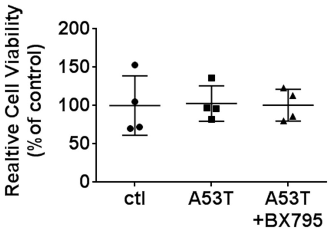

Supplementary Figure 1. Expression of dopaminergic markers in patient p.A53T-iPSC-derived neurons

a. RT-qPCR analysis of selected domaminergic markers in p.A53T iPSC-derived neurons at 21 DIV: Tyrosine Hydrosylase (TH), Nuclear receptor related 1 protein (Nurr1) and Aromatic Lamino acid decarboxylase (AADC) normalized to GAPDH levels. Data represent mean ± SEM (n = 3). Student's t-test was used.

b. Representative images of p.A53T iPSC-derived neurons at 21 DIV immunostained for Ki67 (red) to label cycling cells. Hoechst+ nuclei are in blue (Scale bar, 50 µm). Quantification of the percentage of Ki67+ cells in the presence or absence of BX795. Data represent mean ± SEM (n = 3). Student's t-test was used.

c. Quantification of total nuclei count. Data represent mean ± SEM (n = 4). Comparisons by ANOVA with Tukey correction.

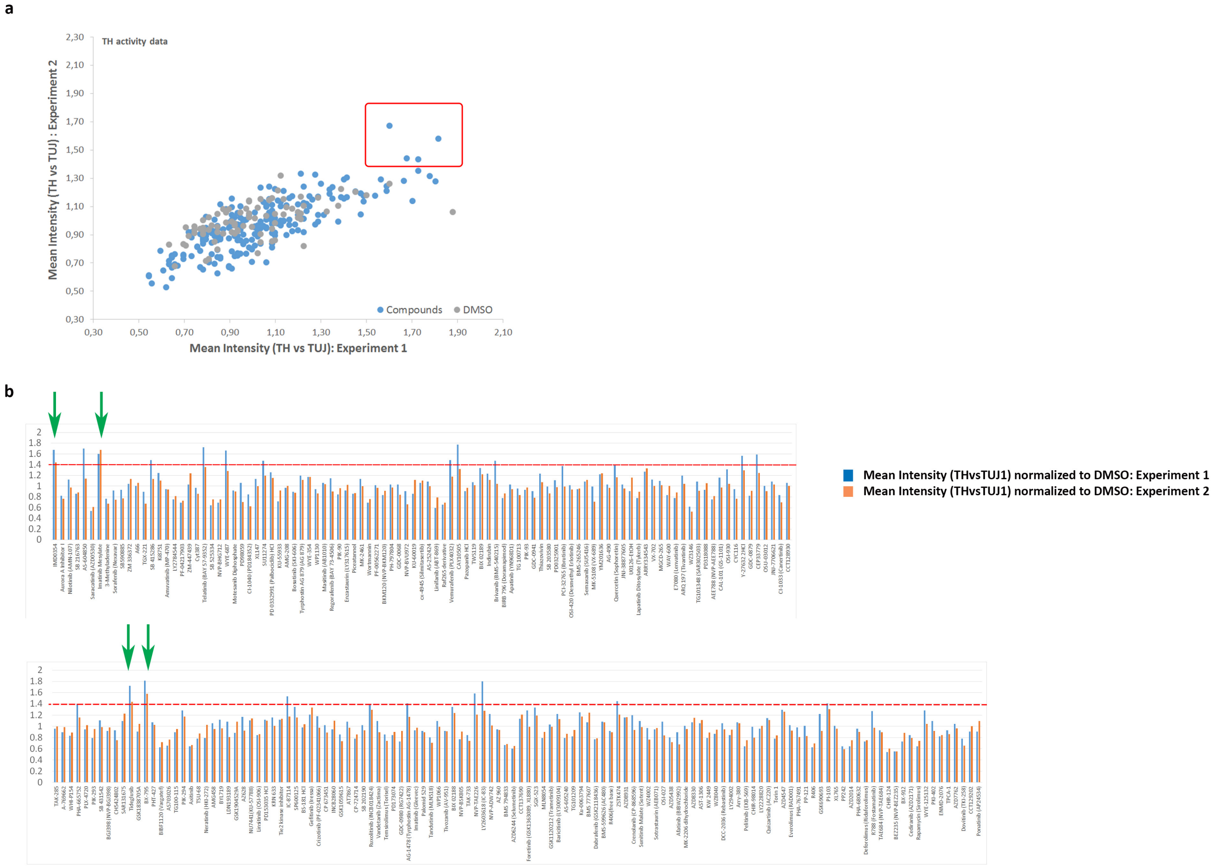

Supplementary Figure 2. High-Content Screening Analysis

a. Scatter plot showing the ratio of TH versus TUJ1 fluorescence intensity in duplicate upon treatment with the small molecule kinase inhibitors (blue dots). The dots inside the rectangle correspond to the 4 hit compounds showing significant increase of TH versus TUJ1 fluorescence ratio as compared to the DMSO controls (grey dots). The toxic compounds have been excluded.

b. Bar graph showing the ratio of TH versus TUJ1 fluorescence intensity that has been normalized to DMSO control for each compound screened. Each color represents one experiment. Horizontal red line is set at two standard deviations and the green arrows show the 4 hit compounds. The toxic compounds have been excluded.

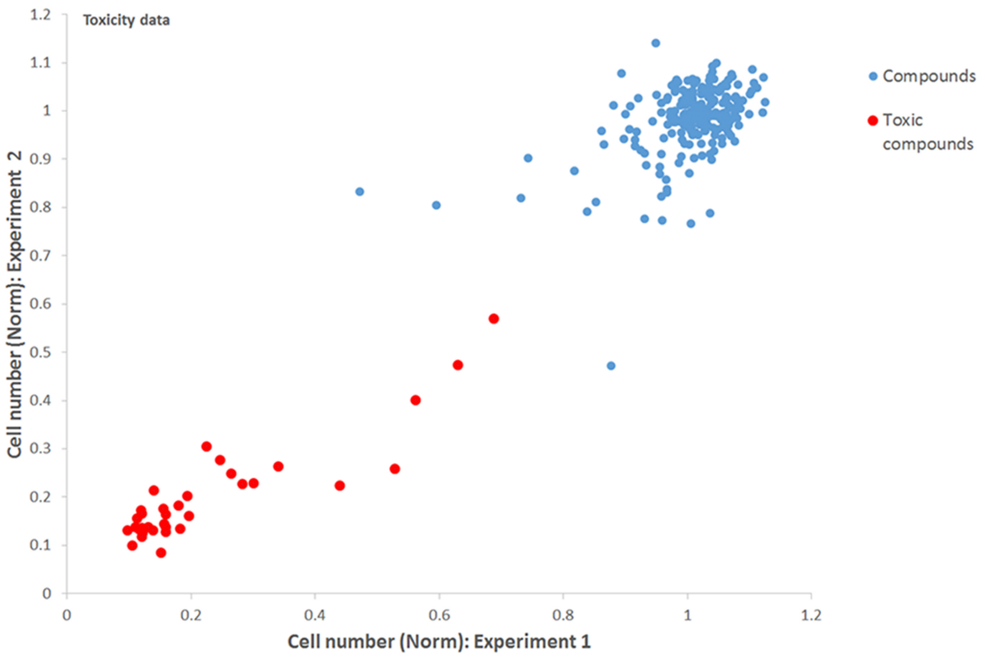

Supplementary Figure 3. Identification of toxic compounds in the small molecule library of kinase inhibitors
Summary of total nuclei counts from two screening plates. Compounds in cells with low nuclei counts were considered toxic and where excluded from the analysis. Each assay plate was normalized to DMSO.

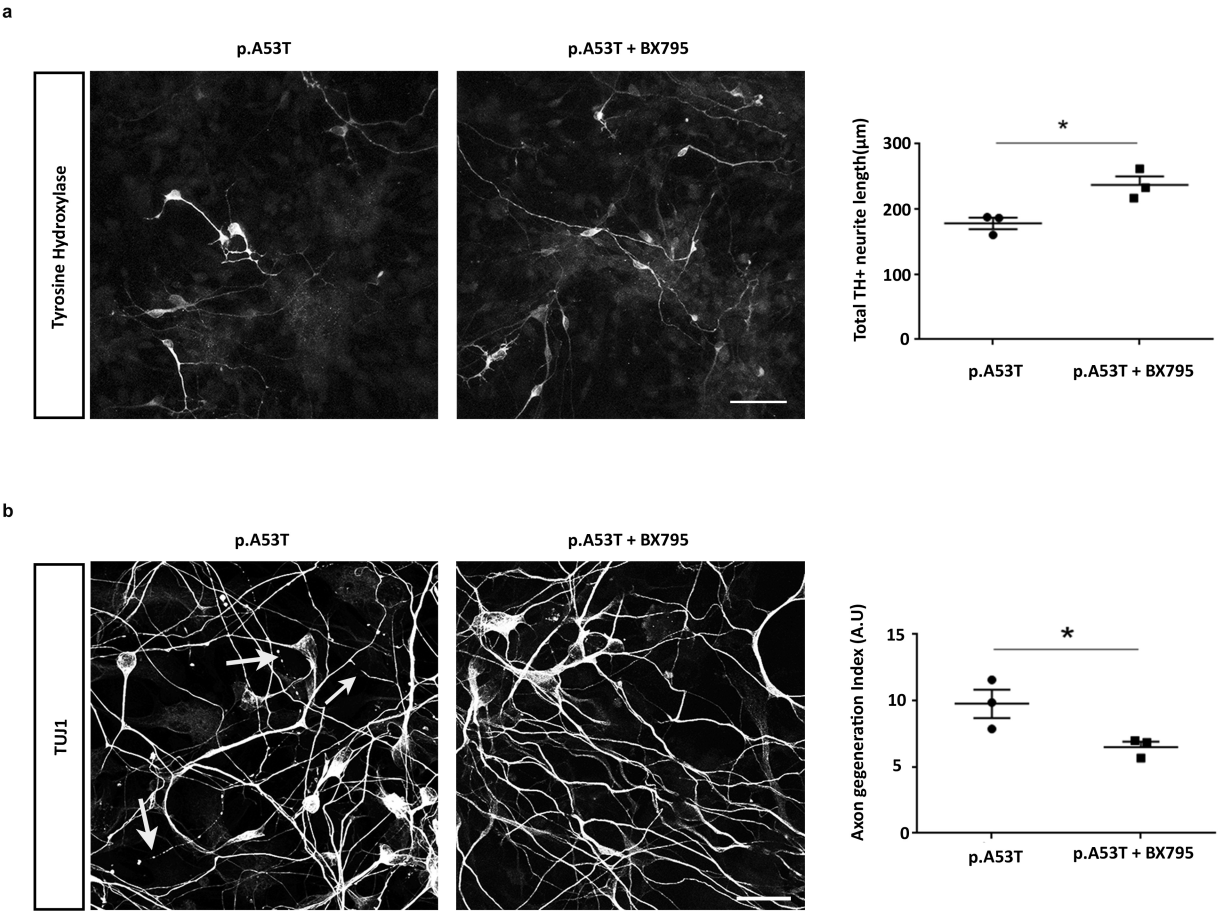

Supplementary Figure 4. Rescue of neuropathological features by BX795 in p.A53T neurons from a second patient

a. BX795 has a positive effect on neurite length of p.A53T-neurons. Representative confocal images of p.A53T-neurons immunostained for TH and quantification of total neurite length of TH+ cells. Data represent mean ± SEM. Student's t-test was used. Scale bar, 50μm.

b. BX795 alleviates axonal neuropathology in p.A53T-neurons as demonstrated by immunostaining for βIII-tubulin (TUJ1; confocal images). Neurites with swollen varicosities or fragmented processes are indicated with arrows. Scale bar, 30μm. Axonal degeneration is estimated in the accompanying graph by measuring the ratio of TUJ1+ spots over the total TUJ1+ area in untreated (DMSO) or BX795-treated p.A53T-neurons. Data represent mean ± SEM. Student's t-test was used.

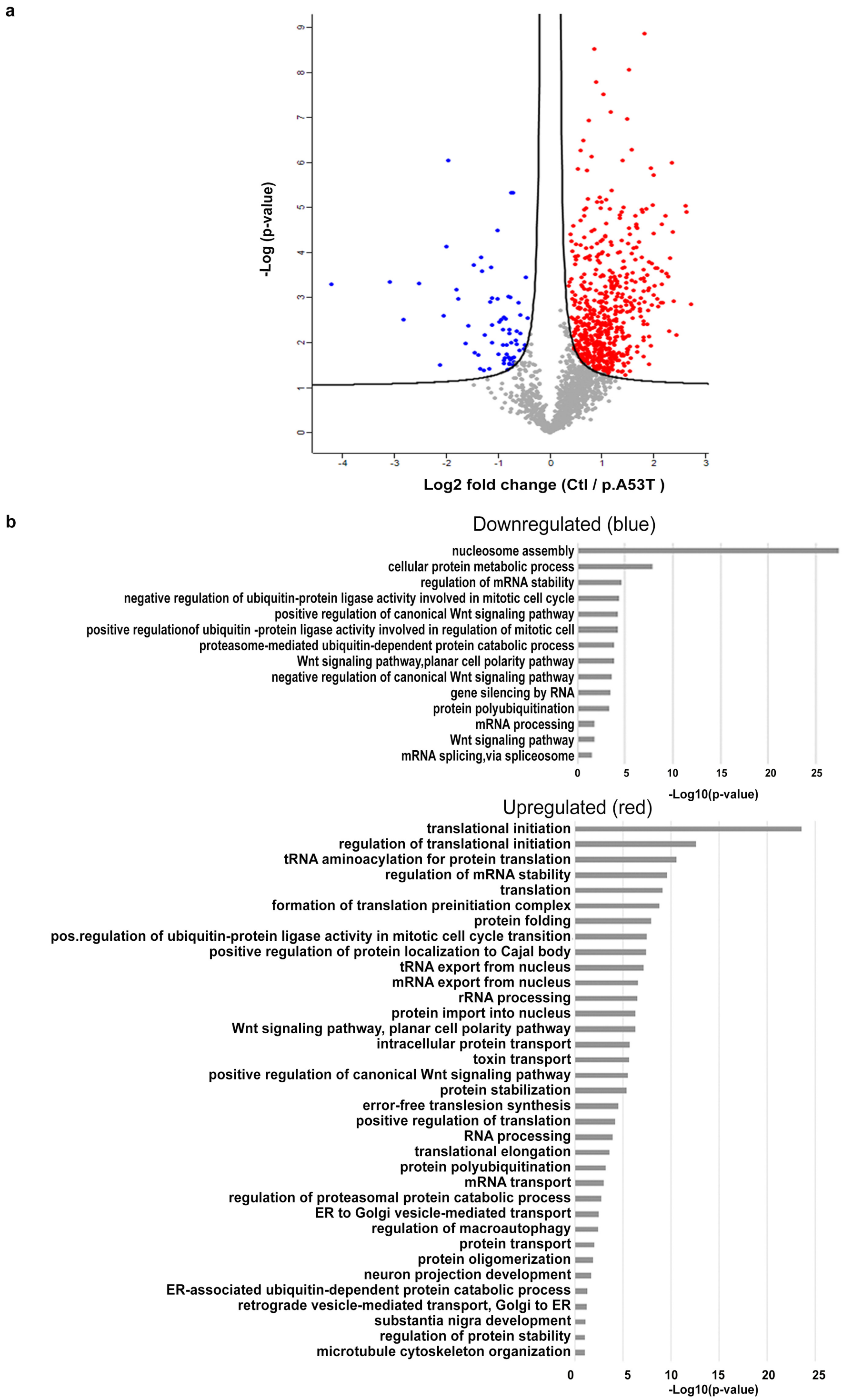

Supplementary Figure 5. Identification of the biological processes that are dysregulated in p.A53T neurons

a. Volcano plot of differentially expressed proteins between control and p.A53T-neurons assessed by quantitative proteomics analysis.Each point represents the difference in expression (fold-change) between the two groups plotted against the level of statistical significance. Blue dots correspond to proteins downregulated in p.A53T neurons while red dots show proteins upregulated in p.A53T neurons (FDR=0.05, S0 = 0.1, as indicated by black lines).

b. GO enrichment analysis for biological processes of the differentially expressed proteins was performed using DAVID software ($p<0.05$).

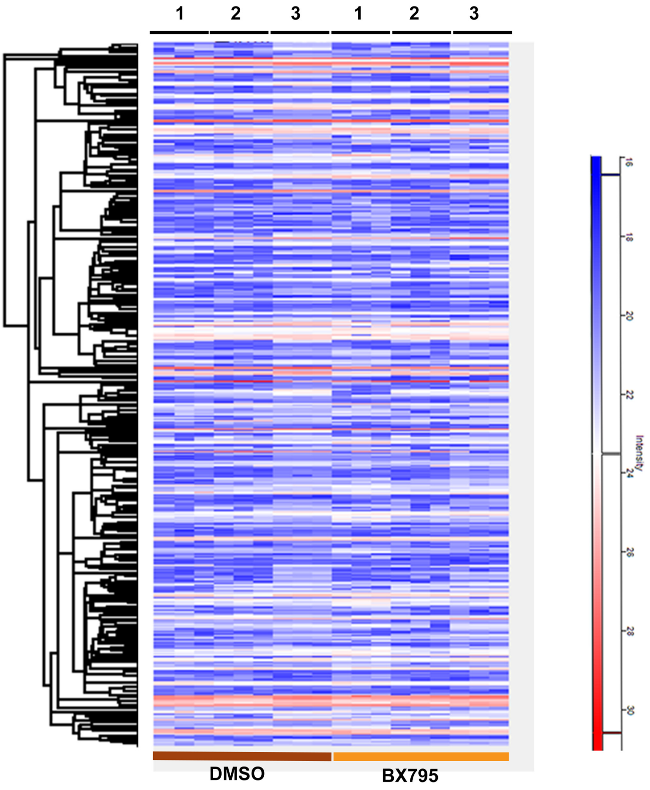

Supplementary Figure 6. Hierarchical clustering of proteins in ctl (WT) cells treated with BX795

Hierarchical clustering shows that there is no change in the proteome of ctl-neurons upon treatment with BX795 (one-way ANOVA analysis). Columns in the different groups (control and crtl-neurons treated with BX795) correspond to individual samples tested and rows represent single proteins (blue, low expression; red, high expression; data are from 3 independent experiments).

Figure 2d

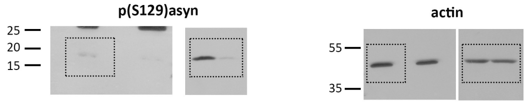

Figure 7a

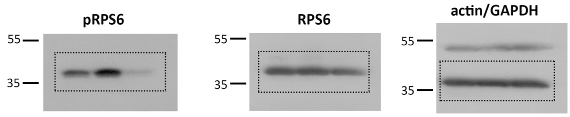

Figure 8b

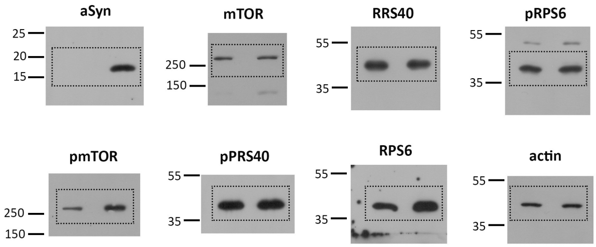

Figure 8c

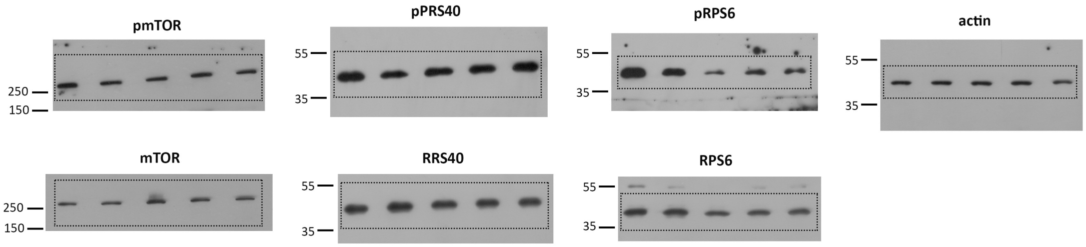

Figure 8e

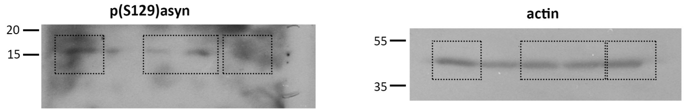

Figure 9b

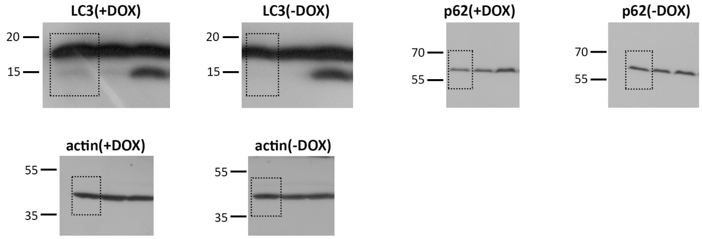

Figure 9e

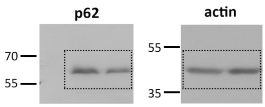

Supplementary Figure 7 . Original unprocessed western blots

Supplementary Table 1. Kinase Inhibitor Library (Selleck Chemicals) and targets

Item Name	Target
Linifanib (ABT-869)	PDGFR, VEGFR
Axitinib	VEGFR, PDGFR, c-Kit
Saracatinib (AZD0530)	Src, Bcr-Abl
AZD6244 (Selumetinib)	MEK
BEZ235 (NVP-BEZ235)	mTOR, PI3K
BIBF1120 (Vargatef)	VEGFR, PDGFR, FGFR
Afatinib (BIBW2992)	EGFR, HER2
Bosutinib (SKI-606)	Src
Cediranib (AZD2171)	VEGFR, Flt
CI-1033 (Canertinib)	EGFR, HER2
CI-1040 (PD184352)	MEK
Dasatinib (BMS-354825)	Src, Bcr-Abl, c-Kit
Deforolimus (Ridaforolimus)	mTOR
Gefitinib (Iressa)	EGFR
Imatinib Mesylate	PDGFR, c-Kit, Bcr-Abl
Lapatinib Ditosylate (Tykerb)	EGFR, HER2
Motesanib Diphosphate	VEGFR, PDGFR, c-Kit
Nilotinib (AMN-107)	Bcr-Abl
Pazopanib HCl	VEGFR, PDGFR, c-Kit
PD0325901	MEK
PI-103	DNA-PK, PI3K, mTOR
Rapamycin (Sirolimus)	mTOR
Sorafenib (Nexavar)	VEGFR, PDGFR, Raf
Sunitinib Malate (Sutent)	VEGFR, PDGFR, c-Kit, Flt
Tandutinib (MLN518)	Flt
Temsirolimus (Torisel)	mTOR
Vandetanib (Zactima)	VEGFR
VX-680 (MK-0457, Tozasertib)	Aurora Kinase
Enzastaurin (LY317615)	PKC
BMS-599626 (AC480)	EGFR, HER2
Masitinib (AB1010)	c-Kit, PDGFR, FGFR, FAK
GDC-0941	PI3K
SB 431542	TGF-beta/Smad
Crizotinib (PF-02341066)	c-Met, ALK
ZSTK474	PI3K
SB 216763	GSK-3
SB 203580	p38 MAPK
SB 202190	p38 MAPK
MK-2206 dihydrochloride	Akt
PD153035 HCl	EGFR
SU11274	c-Met
NVP-ADW742	IGF-1R
KU-55933	ATM
PF-04217903	c-Met
U0126-EtOH	MEK
ZM-447439	Aurora Kinase
GDC-0879	Raf
LY294002	PI3K

Danusertib (PHA-739358)	Aurora Kinase, FGFR, Bcr-Abl, c-RET, Src
TAE684 (NVP-TAE684)	ALK
BI 2536	PLK
Foretinib (GSK1363089, XL880)	c-Met, VEGFR
SGX-523	c-Met
JNJ-38877605	c-Met
PD 0332991 (Palbociclib) HCl	CDK
XL147	PI3K
Everolimus (RAD001)	mTOR
MLN8237 (Alisertib)	Aurora Kinase
AT9283	Bcr-Abl, JAK, Aurora Kinase
AG-490	JAK, EGFR
SNS-032 (BMS-387032)	CDK
Barasertib (AZD1152-HQPA)	Aurora Kinase
PLX-4720	Raf
SNS-314	Aurora Kinase
CP-724714	EGFR, HER2
TGX-221	PI3K
WZ3146	EGFR
CYC116	Aurora Kinase, VEGFR
WZ4002	EGFR
PD98059	MEK
Regorafenib (BAY 73-4506)	c-Kit, Raf, VEGFR
WZ8040	EGFR
ENMD-2076	Flt, Aurora Kinase, VEGFR
PIK-90	PI3K
Tivozanib (AV-951)	VEGFR, c-Kit, PDGFR
OSI-930	c-Kit, VEGFR
Ku-0063794	mTOR
Amuvatinib (MP-470)	c-Met, c-Kit, PDGFR, Flt, c-RET
JNJ-7706621	CDK, Aurora Kinase
WYE-354	mTOR
IC-87114	Others
TG100-115	PI3K
GSK1059615	PI3K, mTOR
MGCD-265	c-Met, VEGFR, Tie-2
Rigosertib (ON-01910)	PLK
Ki8751	VEGFR, c-Kit, PDGFR
Pelitinib (EKB-569)	EGFR
AS-605240	PI3K
Aurora A Inhibitor I	Aurora Kinase
PHA-680632	Aurora Kinase
SP600125	JNK
TSU-68	VEGFR, PDGFR , FGFR
AS703026	MEK
SB 525334	TGF-beta/Smad
HMN-214	PLK
AEE788 (NVP-AEE788)	EGFR, Flt, VEGFR, HER2
PHA-793887	CDK
PIK-93	PI3K, VEGFR

Ponatinib (AP24534)	Bcr-Abl, VEGFR, FGFR, PDGFR, Flt
LY2228820	p38 MAPK
CCT129202	Aurora Kinase
XL765	PI3K, mTOR
AT7519	CDK
Quizartinib (AC220)	Flt
Hesperadin	Aurora Kinase
BIX 02188	MEK
BIX 02189	MEK
AZD7762	Chk
R406(free base)	Syk
AZD8055	mTOR
KRN 633	VEGFR, PDGFR
AT7867	Akt, S6 kinase
BMS 777607	c-Met
PD318088	MEK
KU-60019	ATM
BS-181 HCl	CDK
BIRB 796 (Doramapimod)	p38 MAPK
NVP-BSK805	JAK
DCC-2036 (Rebastinib)	Bcr-Abl
AZD8330	MEK
Neratinib (HKI-272)	HER2, EGFR
KW 2449	Flt, Bcr-Abl, Aurora Kinase
LY2784544	JAK
AZD8931	EGFR, HER2
GSK461364	PLK
R406	Syk, Flt
Raf265 derivative	VEGFR, Raf
BMS 794833	c-Met, VEGFR
NVP-BHG712	VEGFR, Src, Raf, Bcr-Abl
OSI-420 (Desmethyl Erlotinib)	EGFR
PIK-293	PI3K
AZ 960	JAK
Mubritinib (TAK 165)	HER2
PP242	mTOR
Cyt387	JAK
Indirubin	GSK-3
Quercetin (Sophoretin)	PI3K, PKC, Src, Sirtuin
Imatinib (Gleevec)	
GSK2126458	PI3K, mTOR
VX-702	p38 MAPK
CAL-101 (GS-1101)	PI3K
BI6727 (Volasertib)	PLK
PIK-294	PI3K
Telatinib (BAY 57-9352)	VEGFR, PDGFR, c-Kit
AZD5438	CDK
OSI-027	mTOR
PP-121	DNA-PK, mTOR, PDGF
WP1130	DUB, Bcr-Abl

BKM120 (NVP-BKM120)	PI3K
cx-4945 (Silmitasertib)	PKC
LDN193189	TGF-beta/Smad
PF-05212384 (PKI-587)	mTOR, PI3K
TAK-733	MEK
CCT128930	Akt
A66	PI3K
A-674563	Akt, CDK, PKA
AS-252424	PI3K
AS-604850	PI3K
PF-00562271	FAK
WAY-600	mTOR
WYE-125132	mTOR
WYE-687	mTOR
Apatinib (YN968D1)	VEGFR
LY2603618 (IC-83)	Chk
GSK1120212 (Trametinib)	MEK
A-769662	AMPK
KX2-391	Src
PCI-32765 (Ibrutinib)	Src
TAK-901	Aurora Kinase
TG101209	Flt, JAK, c-RET
AMG 900	Aurora Kinase
GSK1838705A	IGF-1, ALK
ZM 336372	Raf
GDC-0980 (RG7422)	mTOR, PI3K
NU7441(KU-57788)	DNA-PK, PI3K
Flavopiridol hydrochloride	CDK
PH-797804	p38 MAPK
Crenolanib (CP-868596)	PDGFR
PF-04691502	mTOR, PI3K, Akt
Dovitinib (TKI-258)	c-Kit, FGFR, Flt, VEGFR, PDGFR
Y-27632 2HCl	ROCK
Brivanib (BMS-540215)	VEGFR, FGFR
GSK1904529A	IGF-1R
MLN8054	Aurora Kinase
OSU-03012	PDK-1
PD173074	FGFR, VEGFR
Vemurafenib (PLX4032)	Raf
AMG-208	c-Met
Thiazovivin	ROCK
Palomid 529	mTOR
PHT-427	Akt, PDK-1
Tie2 kinase inhibitor	Tie-2
Baricitinib (LY3009104)	JAK
E7080 (Lenvatinib)	VEGFR
BGJ398 (NVP-BGJ398)	FGFR
SB590885	Raf
R788 (Fostamatinib)	Syk
CAY10505	PI3K

CHIR-124	Chk
Linsitinib (OSI-906)	IGF-1R
GSK690693	Akt
Ruxolitinib (INCB018424)	JAK
PHA-665752	c-Met
GSK1070916	Aurora Kinase
PKI-402	PI3K
TG101348 (SAR302503)	JAK
PF-03814735	Aurora Kinase, FAK
SB 415286	GSK-3
INK 128	mTOR
Dinaciclib (SCH727965)	CDK
MK-5108 (VX-689)	Aurora Kinase
AG-1478 (Tyrphostin AG-1478)	EGFR
AMG458	c-Met
Arry-380	HER2, EGFR
PHA-848125	CDK
AZ628	Raf
CCT137690	Aurora Kinase
CHIR-98014	GSK-3
NVP-BGT226	PI3K, mTOR
YM201636	PI3K
3-Methyladenine	PI3K
BX-795	PDK-1, IKK
BX-912	PDK-1
CH5424802	ALK
NVP-BVU972	c-Met
AST-1306	EGFR
BMS-265246	CDK
MK-2461	c-Met, FGFR, PDGFR
AZD2014	mTOR
TAK-285	EGFR, HER2
INCB28060	c-Met
WP1066	JAK
Piceatannol	Others
Sotrastaurin (AEB071)	PKC
AZD4547	FGFR
GDC-0068	Akt
Dabrafenib (GSK2118436)	Raf
Tyrphostin AG 879 (AG 879)	HER2
Torin 2	mTOR
BYL719	PI3K
CEP33779	JAK
NVP-TAE226	FAK
CP 673451	Others
PHA-767491	CDK
Torin 1	mTOR
TPCA-1	IKK
Wortmannin	PI3K
Staurosporine	PKC

ARRY334543	EGFR
Tideglusib	GSK-3
Semaxanib (SU5416)	VEGFR
SAR131675	VEGFR
IMD0354	IKK
TG 100713	PI3K
WHI-P154	JAK, EGFR
ARQ 197 (Tivantinib)	c-Met
TWS119	GSK-3

Supplementary Table 2. Differentially expressed proteins between pA53T and control neurons

Protein names	Gene names	Difference	Upregulated	Downregulated
Prothymosin alpha;Prothymosin alpha, N-terminally processed;Thymosin alpha-1	PTMA	-4,22058974		+
Protein transport protein Sec61 subunit alpha isoform 1	SEC61A1	-3,09643152		+
Histone H3.3;Histone H3.2;Histone H3.1t;Histone H3.1	H3f3a;H3F3A;HIST2H3	-2,83120812		+
Fibronectin;Anastellin;Ugl-Y1;Ugl-Y2;Ugl-Y3	FN1	-2,52735837		+
60S ribosomal protein L31	RPL31	-2,12383503		+
Glial fibrillary acidic protein	GFAP	-2,05050193		+
Golgi-associated plant pathogenesis-related protein 1	GLIPR2	-1,997111		+
Histone H1.4	HIST1H1E	-1,96522289		+
Transgelin	TAGLN	-1,80209096		+
Radixin	RDX	-1,77318255		+
Collagen alpha-1(I) chain	COL1A1	-1,63404274		+
Ubiquitin-conjugating enzyme E2 variant 2	UBE2V2	-1,57051065		+
Histone H2A type 2-C;Histone H2A type 2-A	Hist2h2ac;HIST2H2AC	-1,46609285		+
MARCKS-related protein	MARCKSL1	-1,45300293		+
Chromobox protein homolog 1	CBX1	-1,35597377		+
Histone H2B type 1-L;Histone H2B type 1-M;Histone H2B type 1-N;Histone H2B type 1-H;Histone H2B type 1-P;Histone H2B type 1-K;Histone H2B type 1-C/E/G;Histone H2B type 2-B;Histone H2B type 1-B;Histone H2B type 2-F;Histone H2B type 1-C/E/F/G/I;Histone H2B type 1-D;Histone H2B type 1-F/J/L;Histone H2B type F-S;Histone H2B 3;Histone H2B type 1-A	HIST1H2BL;HIST1H2BI	-1,32357407		+
Histone H1.5	HIST1H1B	-1,32074398		+

TAR DNA-binding protein 43	TARDBP	-1,28565174		+
Neutral amino acid transporter A	SLC1A4	-1,26124043		+
Clathrin light chain A	CLTA	-1,16397328		+
Cytoplasmic dynein 1 intermediate chain 2	DYNC1I2;Dync1i2	-1,15378104		+
CDKN2A-interacting protein	CDKN2AIP	-1,13875198		+
Tropomyosin alpha-4 chain	TPM4	-1,1163631		+
Tropomyosin alpha-1 chain	TPM1	-1,11481222		+
Soluble lamin-associated protein of 75 kDa	FAM169A	-1,11439917		+
Ribosome-binding protein 1	RRBP1	-1,01449564		+
Histone H1.2;Histone H1.3	HIST1H1C;Hist1h1d;H	-1,00663524		+
Zinc finger RNA-binding protein	ZFR	-1,00062116		+
Cellular retinoic acid-binding protein 1	CRABP1	-0,97820261		+
45 kDa calcium-binding protein	SDF4	-0,93680615		+
Eukaryotic translation initiation factor 4 gamma 3	EIF4G3	-0,91687775		+
Protein phosphatase 1B	PPM1B	-0,90773561		+
Caldesmon	CALD1	-0,89930958		+
Proteasome subunit alpha type-3	PSMA3	-0,89364327		+
Tubulin beta chain	TUBB	-0,89241261		+
Protein canopy homolog 2	CNPY2	-0,86795319		+
Nuclear pore complex protein Nup153	NUP153	-0,85966153		+
Transmembrane emp24 domain-containing protein 10	TMED10;Tmed10	-0,84096781		+
Rho GTPase-activating protein 21	ARHGAP21	-0,83467611		+
Brain acid soluble protein 1	BASP1	-0,82571665		+
Heterochromatin protein 1-binding protein 3	HP1BP3	-0,81141747		+
Peroxiredoxin-2	PRDX2	-0,80545298		+
Plasma membrane calcium-transporting ATPase 1	ATP2B1	-0,78781933		+
Band 4.1-like protein 3;Band 4.1-like protein 3, N-terminally processed	EPB41L3	-0,78772333		+

Actin, alpha cardiac muscle 1;Actin, alpha skeletal muscle	ACTC1;ACTA1	-0,77967135		+
Polypyrimidine tract-binding protein 2	PTBP2;Ptbp2	-0,77931235		+
Putative phospholipase B-like 2;Putative phospholipase B-like 2 32 kDa form;Putative phospholipase B-like 2 45 kDa form	PLBD2	-0,76606115		+
Heterogeneous nuclear ribonucleoprotein A1;Heterogeneous nuclear ribonucleoprotein A1, N-terminally processed;Heterogeneous nuclear ribonucleoprotein A1-like 2	HNRNPA1;HNRNPA1L	-0,76263936		+
Acyl-CoA dehydrogenase family member 9, mitochondrial	ACAD9	-0,7569809		+
Kinectin	KTN1	-0,74694803		+
UBX domain-containing protein 1	UBXN1	-0,72750621		+
ATP synthase subunit d, mitochondrial	ATP5H	-0,72205056		+
Reticulon-4	RTN4	-0,71967845		+
Amyloid beta A4 protein;N-APP;Soluble APP-alpha;Soluble APP-beta;C99;Beta-amyloid protein 42;Beta-amyloid protein 40;C83;P3(42);P3(40);C80;Gamma-secretase C-terminal fragment 59;Gamma-secretase C-terminal fragment 57;Gamma-secretase C-terminal fragment 50;C31	APP;App	-0,70541975		+
Formin-binding protein 1-like	FNBP1L	-0,69941372		+
Ubiquitin-40S ribosomal protein S27a;Ubiquitin;40S ribosomal protein S27a;Ubiquitin-60S ribosomal protein L40;Ubiquitin;60S ribosomal protein L40;Polyubiquitin-B;Ubiquitin;Polyubiquitin-C;Ubiquitin;Polyubiquitin-A;Ubiquitin;Ubiquitin-related	RPS27A;Rps27a;UBA5	-0,66847505		+

Prelamin-A/C;Lamin-A/C	LMNA	-0,66254658		+
N(G),N(G)-dimethylarginine dimethylaminohydrolase 2	DDAH2	-0,64632734		+
U4/U6.U5 tri-snRNP-associated protein 1	SART1;Sart1	-0,63747215		+
Splicing factor, proline- and glutamine-rich	SFPQ	-0,61198764		+
Delta-1-pyrroline-5-carboxylate dehydrogenase, mitochondrial	ALDH4A1	-0,58621025		+
Pre-mRNA-processing factor 40 homolog A	PRPF40A	-0,57962841		+
Zyxin	ZYX	-0,56851408		+
Histone H4	Hist1h4a;HIST1H4A	-0,49797291		+
Catenin delta-1	CTNND1	-0,48542023		+
Lamin-B1	LMNB1	-0,47633659		+
Reticulocalbin-1	RCN1	-0,44097794		+
Polyadenylate-binding protein 1	PABPC1	0,36678696	+	
T-complex protein 1 subunit eta	CCT7	0,37164391	+	
ELAV-like protein 1	ELAVL1	0,38943545	+	
Nitric oxide synthase-interacting protein	NOSIP	0,40467771	+	
Lamina-associated polypeptide 2, isoform alpha;Thymopoietin;Thymopentin	TMPO	0,41073651	+	
Chromobox protein homolog 5	CBX5;Cbx5	0,41383574	+	
Elongation factor 2	EEF2;Eef2	0,42066256	+	
60S ribosomal protein L4	RPL4	0,4245472	+	
Cell cycle and apoptosis regulator protein 2	CCAR2	0,43223847	+	
Pre-mRNA-splicing factor ATP-dependent RNA helicase DHX15	DHX15	0,44824028	+	
Ubiquitin thioesterase OTUB1	OTUB1	0,44847086	+	
Myosin-10	MYH10	0,44919946	+	
Platelet-activating factor acetylhydrolase IB subunit alpha	PAFAH1B1;Pafah1b1	0,46440866	+	
MICOS complex subunit MIC60	IMMT	0,47223282	+	
General transcription factor II-I	GTF2I;Gtf2i	0,47717624	+	

Voltage-dependent anion-selective channel protein 2	VDAC2	0,47729916	+	
DNA topoisomerase 1	TOP1	0,47934278	+	
60 kDa heat shock protein, mitochondrial	HSPD1	0,48447143	+	
Far upstream element-binding protein 2	KHSRP	0,48472828	+	
Stress-70 protein, mitochondrial	HSPA9	0,48504872	+	
Dynamin-2	DNM2	0,48548105	+	
Hydroxymethylglutaryl-CoA synthase, cytoplasmic	HMGCS1	0,48664877	+	
Sarcoplasmic/endoplasmic reticulum calcium ATPase 2	ATP2A2	0,49363581	+	
Multifunctional protein ADE2;Phosphoribosylaminoimidazole-succinocarboxamide synthase;Phosphoribosylaminoimidazole carboxylase	PAICS	0,49574788	+	
Bifunctional purine biosynthesis protein PURH;Phosphoribosylaminoimidazolecarboxamide formyltransferase;IMP cyclohydrolase	ATIC	0,49794049	+	
Isocitrate dehydrogenase [NADP], mitochondrial	IDH2	0,49905989	+	
Adenosylhomocysteinase	AHCY	0,50178507	+	
Methionine--tRNA ligase, cytoplasmic	MARS	0,50235918	+	
Eukaryotic initiation factor 4A-III;Eukaryotic initiation factor 4A-III, N-terminally processed	EIF4A3	0,50351991	+	
Coatomer subunit alpha;Xenin;Proxenin	COPA	0,50397809	+	
Rab GDP dissociation inhibitor beta	GDI2	0,5050727	+	
Oxysterol-binding protein 1	OSBP	0,50976329	+	
Ras-related protein Rab-2A	RAB2A	0,51790598	+	
NADPH--cytochrome P450 reductase	POR	0,52251604	+	
Aspartate aminotransferase, mitochondrial	GOT2	0,52831353	+	

Eukaryotic translation initiation factor 3 subunit E	EIF3E	0,53256332	+	
40S ribosomal protein SA	Rpsa;RPSA	0,53590287	+	
Stress-induced-phosphoprotein 1	STIP1	0,53696569	+	
Serine-threonine kinase receptor-associated protein	STRAP	0,53877873	+	
2,4-dienoyl-CoA reductase, mitochondrial	DECR1	0,54022598	+	
Malate dehydrogenase, mitochondrial	MDH2	0,54088974	+	
Malectin	MLEC	0,54325846	+	
Coatomer subunit beta	COPB2	0,54395019	+	
Septin-9	SEPT9	0,54734124	+	
CTP synthase 1	CTPS1	0,55151049	+	
Signal transducer and activator of transcription 3	STAT3	0,55413225	+	
Cysteine protease ATG4B	ATG4B;Atg4b	0,55785137	+	
Flap endonuclease 1	FEN1	0,56196107	+	
ATP-dependent RNA helicase DDX19A;ATP-dependent RNA helicase DDX19B	DDX19A;DDX19B	0,56328519	+	
Nuclear pore membrane glycoprotein 210	NUP210	0,56637213	+	
Heat shock 70 kDa protein 4	HSPA4	0,566942	+	
Phosphoserine aminotransferase	PSAT1	0,57374679	+	
Pyruvate dehydrogenase E1 component subunit beta, mitochondrial	PDHB	0,57605701	+	
Transitional endoplasmic reticulum ATPase	Vcp;VCP	0,58135562	+	
Arginine--tRNA ligase, cytoplasmic	RARS	0,58195093	+	
X-ray repair cross-complementing protein 6	XRCC6	0,58255429	+	
Bifunctional glutamate/proline--tRNA ligase;Glutamate--tRNA ligase;Proline--tRNA ligase	EPRS	0,58510844	+	
Peroxiredoxin-6	PRDX6	0,58962144	+	

CAD protein;Glutamine-dependent carbamoyl-phosphate synthase;Aspartate carbamoyltransferase;Dihydroorotase	CAD	0,59100299	+	
Farnesyl pyrophosphate synthase	FDPS	0,59512181	+	
Cyclin-dependent-like kinase 5	CDK5	0,60137918	+	
Ran GTPase-activating protein 1	RANGAP1	0,60225423	+	
Heterogeneous nuclear ribonucleoprotein D-like	HNRNPDL	0,60320918	+	
NEDD8-activating enzyme E1 catalytic subunit	UBA3	0,60380046	+	
T-complex protein 1 subunit beta	CCT2	0,60664368	+	
Cytoskeleton-associated protein 5	CKAP5	0,60961533	+	
Prohibitin	PHB	0,61144045	+	
Unconventional myosin-VI	MYO6	0,61296166	+	
Cleavage stimulation factor subunit 3	CSTF3	0,61377652	+	
Importin-5	IPO5	0,61648496	+	
Very-long-chain (3R)-3-hydroxyacyl-CoA dehydratase 3	HACD3	0,61995697	+	
Glycine--tRNA ligase	GARS	0,62528377	+	
Thioredoxin-like protein 1	TXNL1	0,62663396	+	
Isoleucine--tRNA ligase, cytoplasmic	IARS	0,62683529	+	
Pyruvate carboxylase, mitochondrial	PC	0,63483853	+	
T-complex protein 1 subunit gamma	CCT3	0,6354794	+	
40S ribosomal protein S10;Putative 40S ribosomal protein S10-like	RPS10;RPS10P5	0,63819567	+	
182 kDa tankyrase-1-binding protein	TNKS1BP1	0,64544317	+	
ATP-citrate synthase	ACLY;Acly	0,64815733	+	
Inositol-3-phosphate synthase 1	ISYNA1	0,65151003	+	
Guanine nucleotide-binding protein subunit beta-2-like 1;Guanine nucleotide-binding protein subunit beta-2-like 1, N-terminally processed	GNB2L1	0,6532597	+	
Probable ATP-dependent RNA helicase DDX6;ATP-dependent RNA helicase ddx6	DDX6;Ddx6;ddx6	0,65331353	+	

Gem-associated protein 5	GEMIN5	0,65372573	+	
Mitochondrial-processing peptidase subunit alpha	PMPCA	0,65491528	+	
ATP-dependent RNA helicase DDX1	DDX1	0,65614933	+	
Neurofilament light polypeptide	NEFL	0,65656302	+	
40S ribosomal protein S6	RPS6	0,65667152	+	
Methylmalonate-semialdehyde dehydrogenase [acylating], mitochondrial	ALDH6A1	0,65771061	+	
Heterogeneous nuclear ribonucleoprotein L	HNRNPL	0,65942128	+	
Exportin-1	XPO1;Xpo1	0,66231028	+	
T-complex protein 1 subunit theta	CCT8	0,66265233	+	
Heterogeneous nuclear ribonucleoprotein M	HNRNPM	0,66267946	+	
T-complex protein 1 subunit epsilon	CCT5	0,66413583	+	
Lysine--tRNA ligase	KARS	0,66436916	+	
Carboxymethylenebutenolidase homolog	CMBL	0,66666497	+	
Uncharacterized protein C7orf50	C7orf50	0,66734335	+	
Neurofilament medium polypeptide	NEFM	0,6674739	+	
Dynamin-1-like protein	DNM1L	0,66997189	+	
Nuclear pore complex protein Nup50	NUP50	0,67132717	+	
Peroxiredoxin-1	PRDX1	0,67464023	+	
Ubiquitin carboxyl-terminal hydrolase 5	USP5	0,67514716	+	
Chloride intracellular channel protein 1	CLIC1	0,67566193	+	
T-complex protein 1 subunit delta	CCT4	0,6770969	+	
Elongation factor 1-beta	EEF1B2	0,67718993	+	
AP-2 complex subunit alpha-2	AP2A2	0,68129052	+	
Septin-2	SEPT2	0,68638208	+	
High mobility group protein B1;Putative high mobility group protein B1-like 1	HMGB1;Hmgb1;HMG	0,6891399	+	
Replication factor C subunit 1	RFC1	0,69490157	+	
MICOS complex subunit MIC19	CHCHD3	0,69938045	+	
Alpha-internexin	INA	0,7001809	+	

RNA polymerase II-associated factor 1 homolog	PAF1	0,70027139	+	
60S ribosomal protein L5	RPL5	0,7005859	+	
Sideroflexin-1	SFXN1	0,701732	+	
Poly(rC)-binding protein 2	PCBP2	0,70254792	+	
Protein crumbs homolog 2	CRB2	0,70587688	+	
39S ribosomal protein L22, mitochondrial	MRPL22	0,70988634	+	
Cytoskeleton-associated protein 4	CKAP4	0,70989778	+	
Tumor protein D54	TPD52L2	0,71015655	+	
Metastasis-associated protein MTA2	MTA2;Mta2	0,71304385	+	
mRNA cap guanine-N7 methyltransferase	RNMT	0,71329859	+	
Ubiquitin carboxyl-terminal hydrolase isozyme L1	UCHL1	0,71483188	+	
Cytochrome b-c1 complex subunit 2, mitochondrial	UQCRC2	0,715438	+	
YLP motif-containing protein 1	YLPM1	0,71668392	+	
ATP-dependent DNA helicase Q1	RECQL	0,71730084	+	
Vacuolar protein sorting-associated protein 35	VPS35;Vps35	0,71786329	+	
Chromobox protein homolog 3	CBX3	0,71830495	+	
60S ribosomal protein L12	RPL12	0,72048357	+	
Sodium/potassium-transporting ATPase subunit beta-1	ATP1B1	0,72105302	+	
Protein LSM14 homolog B	LSM14B	0,72187487	+	
C-1-tetrahydrofolate synthase, cytoplasmic;Methylenetetrahydrofolate dehydrogenase;Methenyltetrahydrofolate cyclohydrolase;Formyltetrahydrofolate synthetase;C-1-tetrahydrofolate synthase, cytoplasmic, N-terminally processed	MTHFD1	0,72321616	+	
40S ribosomal protein S25	RPS25	0,72731739	+	
FACT complex subunit SSRP1	SSRP1	0,72842047	+	
Huntingtin-interacting protein 1	HIP1	0,72859001	+	

Pyrroline-5-carboxylate reductase 1, mitochondrial	PYCR1	0,72987959	+	
Pre-mRNA-processing-splicing factor 8	PRPF8	0,7321896	+	
HBS1-like protein	HBS1L	0,73350504	+	
Adenylosuccinate synthetase isozyme 2	ADSS	0,73512416	+	
Protein arginine N-methyltransferase 1	PRMT1	0,7378042	+	
Alanine--tRNA ligase, cytoplasmic	AARS	0,73953523	+	
O-acetyl-ADP-ribose deacetylase 1	OARD1	0,74088754	+	
SUMO-activating enzyme subunit 2	UBA2	0,74310282	+	
Nuclear pore complex protein Nup205	NUP205	0,74427859	+	
Ataxin-10	ATXN10	0,75490401	+	
Glutathione peroxidase 1	GPX1	0,75532087	+	
Calcyclin-binding protein	CACYBP	0,75540161	+	
F-actin-capping protein subunit beta	CAPZB	0,75865576	+	
Alpha-aminoadipic semialdehyde dehydrogenase	ALDH7A1	0,76025094	+	
Calcium-binding mitochondrial carrier protein Aralar1	SLC25A12	0,76159901	+	
Aconitate hydratase, mitochondrial	ACO2	0,76345507	+	
Sorting nexin-6;Sorting nexin-6, N-terminally processed	SNX6	0,76497332	+	
Heat shock 70 kDa protein 12A	HSPA12A	0,76612684	+	
Basic leucine zipper and W2 domain-containing protein 1	BZW1	0,77025922	+	
Enhancer of mRNA-decapping protein 4	EDC4	0,78210153	+	
Nuclear protein localization protein 4 homolog	NPLOC4	0,78391669	+	
Tyrosine-protein kinase CSK	CSK	0,78742769	+	
Translation initiation factor eIF-2B subunit delta	EIF2B4	0,78922335	+	
E3 ubiquitin-protein ligase UHRF1	UHRF1	0,78940964	+	
60S ribosomal protein L27a	RPL27A;Rpl27a	0,79095183	+	
26S proteasome non-ATPase regulatory subunit 4	PSMD4	0,79130618	+	

FACT complex subunit SPT16	SUPT16H	0,79339176	+	
Trifunctional purine biosynthetic protein adenosine-3;Phosphoribosylamine--glycine ligase;Phosphoribosylformylglycinamidine cyclo-ligase;Phosphoribosylglycinamide formyltransferase	GART	0,79848162	+	
Eukaryotic translation initiation factor 4 gamma 2	EIF4G2	0,80582746	+	
E2/E3 hybrid ubiquitin-protein ligase UBE2O	UBE2O	0,80629285	+	
Eukaryotic translation initiation factor 3 subunit C;Eukaryotic translation initiation factor 3 subunit C-like protein	EIF3C;EIF3CL	0,80635749	+	
DNA ligase 3	LIG3	0,80947198	+	
ATP-binding cassette sub-family F member 1	ABCF1	0,81026226	+	
Nuclear pore complex protein Nup88	NUP88	0,8166737	+	
Adipocyte plasma membrane-associated protein	APMAP	0,81971105	+	
Cysteine and glycine-rich protein 1	CSRP1	0,8229582	+	
Nucleoporin p54	NUP54	0,82570903	+	
ATP-dependent 6-phosphofructokinase, muscle type	PFKM	0,83510272	+	
Protein kinase C and casein kinase substrate in neurons protein 2	PACSIN2	0,83510399	+	
DNA repair protein XRCC1	XRCC1	0,83597565	+	
Succinate-semialdehyde dehydrogenase, mitochondrial	ALDH5A1	0,83830134	+	
Aflatoxin B1 aldehyde reductase member 2	AKR7A2	0,8383984	+	
E3 ubiquitin-protein ligase BRE1A	RNF20	0,83881548	+	
PHD finger protein 6	PHF6	0,8417937	+	
28S ribosomal protein S31, mitochondrial	MRPS31	0,8446863	+	

Probable global transcription activator SNF2L1	SMARCA1;Smarca1	0,84491963	+	
Regulator of chromosome condensation	RCC1	0,84653388	+	
Cyclin-dependent kinase 11A;Cyclin-dependent kinase 11B	CDK11A;CDK11B	0,84698232	+	
39S ribosomal protein L1, mitochondrial	MRPL1	0,84698423	+	
Pinin	PNN	0,8496774	+	
Fatty acid synthase;[Acyl-carrier-protein] S-acetyltransferase;[Acyl-carrier-protein] S-malonyltransferase;3-oxoacyl-[acyl-carrier-protein] synthase;3-oxoacyl-[acyl-carrier-protein] reductase;3-hydroxyacyl-[acyl-carrier-protein] dehydratase;Enoyl-[acyl-carrier-protein] reductase;Oleoyl-[acyl-carrier-protein] hydrolase	FASN	0,85016759	+	
Protein transport protein Sec31A	SEC31A	0,85054546	+	
Structural maintenance of chromosomes protein 1A	SMC1A;smc1a	0,85207791	+	
Protein transport protein Sec23A	SEC23A	0,85275926	+	
Kinesin-like protein KIF1A	KIF1A	0,8551178	+	
Squalene synthase	FDFT1	0,85576375	+	
Eukaryotic translation initiation factor 2 subunit 3;Putative eukaryotic translation initiation factor 2 subunit 3-like protein;Eukaryotic translation initiation factor 2 subunit 3, X-linked;Eukaryotic translation initiation factor 2 subunit 3, Y-linked	EIF2S3;Eif2s3;EIF2S3L	0,85691494	+	
Heterogeneous nuclear ribonucleoprotein F;Heterogeneous nuclear ribonucleoprotein F, N-terminally processed	HNRNPF	0,85778597	+	
Squalene monooxygenase	SQLE	0,85792033	+	
T-complex protein 1 subunit alpha	TCP1;Tcp1	0,86092822	+	

10 kDa heat shock protein, mitochondrial	HSPE1	0,86157121	+	
Quinone oxidoreductase	CRYZ	0,86160787	+	
Glycylpeptide N-tetradecanoyltransferase 1	NMT1	0,87303861	+	
Glutaredoxin-3	GLRX3	0,8834112	+	
Heat shock protein 105 kDa	HSPH1	0,88477092	+	
Probable glutathione peroxidase 8	GPX8	0,88546732	+	
Intraflagellar transport protein 27 homolog	IFT27	0,88639641	+	
Protein O-GlcNAcase	MGEA5;Mgea5	0,8876614	+	
Importin-4	IPO4	0,8891076	+	
Exosome complex component RRP43	EXOSC8	0,89599164	+	
Protein DEK	DEK	0,89820035	+	
ADP-ribosylation factor-like protein 8A	ARL8A	0,89923392	+	
Synaptobrevin homolog YKT6	YKT6	0,89995914	+	
Eukaryotic initiation factor 4A-I	EIF4A1	0,90034993	+	
N-acetylserotonin O-methyltransferase-like protein	ASMTL	0,90054046	+	
Histidine--tRNA ligase, cytoplasmic	HARS	0,90092129	+	
Glutamine--tRNA ligase	QARS	0,90180757	+	
Apoptosis regulator BAX	BAX	0,90222761	+	
Cysteine and histidine-rich domain-containing protein 1	CHORDC1	0,90228123	+	
Calcium-binding mitochondrial carrier protein Aralar2	SLC25A13	0,90638924	+	
Atlastin-1	ATL1	0,90722953	+	
26S proteasome non-ATPase regulatory subunit 8	PSMD8	0,90734715	+	
Uridine 5-monophosphate synthase;Orotate phosphoribosyltransferase;Orotidine 5-phosphate decarboxylase	UMPS	0,91802639	+	
Ras-related protein Rab-18	RAB18	0,92116186	+	

26S proteasome non-ATPase regulatory subunit 10	PSMD10	0,921548	+	
Lanosterol 14-alpha demethylase	CYP51A1	0,92195278	+	
Membrane-associated progesterone receptor component 2	PGRMC2	0,92723952	+	
THO complex subunit 4	ALYREF	0,92735121	+	
Filamin-C	FLNC	0,92777104	+	
Valine--tRNA ligase	VARS	0,92905405	+	
Cleavage and polyadenylation specificity factor subunit 5	NUDT21	0,93198013	+	
Heterogeneous nuclear ribonucleoprotein U-like protein 1	HNRNPUL1	0,93243514	+	
Eukaryotic translation initiation factor 3 subunit F	EIF3F	0,93482166	+	
Septin-5	SEPT5	0,93491491	+	
Eukaryotic translation initiation factor 3 subunit H	EIF3H	0,9407408	+	
NEDD8-activating enzyme E1 regulatory subunit	NAE1	0,94177945	+	
Sideroflexin-3	SFXN3	0,94284206	+	
Alcohol dehydrogenase class-3	ADH5	0,94287194	+	
Dolichyl-diphosphooligosaccharide--protein glycosyltransferase subunit STT3B	STT3B	0,94328859	+	
40S ribosomal protein S9	RPS9	0,945062	+	
Poly(rC)-binding protein 1	PCBP1	0,94605361	+	
Nuclear pore complex protein Nup85	NUP85	0,95299551	+	
Lysine-specific histone demethylase 1A	KDM1A	0,95578766	+	
Sister chromatid cohesion protein PDS5 homolog B	PDS5B	0,95589892	+	
26S proteasome non-ATPase regulatory subunit 11	PSMD11	0,95619117	+	
Histone acetyltransferase type B catalytic subunit	HAT1	0,95839225	+	
Nuclear pore complex protein Nup155	NUP155;Nup155	0,95919906	+	

Vacuolar protein sorting-associated protein 4B	VPS4B	0,95933194	+	
Eukaryotic translation initiation factor 3 subunit M	EIF3M	0,95983484	+	
Spermine synthase	SMS	0,95988231	+	
Actin-related protein 2;Actin-related protein 2-B	ACTR2;actr2b	0,96049881	+	
Adenine phosphoribosyltransferase	APRT	0,96247821	+	
Ankycorbin	RAI14	0,96523158	+	
Transmembrane protein 33	TMEM33	0,96590932	+	
40S ribosomal protein S3	RPS3	0,96805848	+	
Eukaryotic translation initiation factor 3 subunit B	EIF3B	0,96913613	+	
Myosin regulatory light chain 12A;Myosin regulatory light chain 12B	MYL12A;MYL12B	0,97154744	+	
Eukaryotic translation initiation factor 3 subunit D	EIF3D	0,97200288	+	
Protein phosphatase methylesterase 1	PPME1	0,9724297	+	
DNA replication licensing factor MCM6	MCM6	0,97616132	+	
Anamorsin	CIAPIN1	0,9777209	+	
ADP-sugar pyrophosphatase	NUDT5	0,97962591	+	
Cullin-1	Cul1;CUL1	0,98065461	+	
Splicing factor 45	RBM17	0,98133341	+	
Syntaxin-12	STX12	0,9816316	+	
Hsc70-interacting protein;Putative protein FAM10A5;Putative protein FAM10A4	ST13;ST13P5;ST13P4	0,98399353	+	
V-type proton ATPase subunit d 1	ATP6V0D1	0,98763635	+	
Methionine adenosyltransferase 2 subunit beta	MAT2B	0,98799621	+	
Probable ATP-dependent RNA helicase DDX5	DDX5	0,9885966	+	
Nucleoside diphosphate kinase B	NME2	0,98965306	+	

Mycophenolic acid acyl-glucuronide esterase, mitochondrial	ABHD10	0,99094348	+	
Ubiquitin-conjugating enzyme E2 E3;Ubiquitin-conjugating enzyme E2 E2	UBE2E3;Ube2e3;UBE2	0,99097697	+	
Nuclear pore complex protein Nup133	NUP133	0,99205356	+	
5-3 exoribonuclease 2	XRN2	0,99258635	+	
GDP-L-fucose synthase	TSTA3	0,99589411	+	
Syntenin-1	SDCBP	0,99601788	+	
U2 snRNP-associated SURP motif-containing protein	U2SURP	0,99694697	+	
UDP-N-acetylglucosamine--peptide N-acetylglucosaminyltransferase 110 kDa subunit	OGT	0,99701733	+	
Proteasome subunit alpha type-1	PSMA1	0,99923706	+	
Myosin light polypeptide 6	MYL6	0,99941953	+	
Condensin complex subunit 3	NCAPG	1,00285445	+	
Agrin;Agrin N-terminal 110 kDa subunit;Agrin C-terminal 110 kDa subunit;Agrin C-terminal 90 kDa fragment;Agrin C-terminal 22 kDa fragment	AGRN	1,00335926	+	
Exportin-2	CSE1L	1,00356144	+	
Fanconi anemia group I protein	FANCI	1,00651783	+	
Isopentenyl-diphosphate Delta-isomerase 1	IDI1	1,0073897	+	
Mitochondrial import receptor subunit TOM70	TOMM70A	1,00825564	+	
Selenide, water dikinase 1	SEPHS1	1,0087293	+	
Synaptic vesicle membrane protein VAT-1 homolog-like	VAT1L	1,01057137	+	
Laminin subunit gamma-1	LAMC1	1,0107265	+	
DnaJ homolog subfamily C member 7	DNAJC7	1,01387151	+	
Nucleolar GTP-binding protein 1	GTPBP4	1,01682303	+	

Mesencephalic astrocyte-derived neurotrophic factor	MANF	1,02139431	+	
Proliferating cell nuclear antigen	PCNA	1,02184698	+	
Signal recognition particle receptor subunit beta	SRPRB	1,0224639	+	
Casein kinase II subunit alpha	CSNK2A2	1,0233061	+	
Ephrin type-A receptor 2	EPHA2	1,02584394	+	
Ubiquitin-conjugating enzyme E2 K	UBE2K	1,03290727	+	
ELAV-like protein 3	ELAVL3	1,03751861	+	
Apoptosis inhibitor 5	API5	1,03793229	+	
Peptidyl-prolyl cis-trans isomerase FKBP10	FKBP10	1,04088169	+	
Translocon-associated protein subunit delta	SSR4	1,04090563	+	
AP-3 complex subunit delta-1	AP3D1	1,04234166	+	
Ubiquitin carboxyl-terminal hydrolase 14	USP14	1,04278119	+	
28 kDa heat- and acid-stable phosphoprotein	PDAP1	1,04491382	+	
Serine/threonine-protein phosphatase 2A catalytic subunit alpha isoform	PPP2CA	1,04501343	+	
NADH dehydrogenase [ubiquinone] iron-sulfur protein 2, mitochondrial	NDUFS2	1,04504479	+	
Electron transfer flavoprotein subunit beta	ETFB	1,04917781	+	
NADH dehydrogenase [ubiquinone] iron-sulfur protein 3, mitochondrial	NDUFS3	1,05075328	+	
Serine/threonine-protein phosphatase 2A activator	PPP2R4	1,0543357	+	
Golgi-specific brefeldin A-resistance guanine nucleotide exchange factor 1	GBF1	1,05519549	+	
Acetyl-CoA carboxylase 1;Biotin carboxylase	ACACA	1,05589909	+	
Replication factor C subunit 5	RFC5	1,05746905	+	

NAD-dependent malic enzyme, mitochondrial	ME2	1,06008848	+	
Protein PBDC1	PBDC1	1,06041993	+	
Regulator of nonsense transcripts 1	UPF1	1,0617307	+	
Methylmalonyl-CoA mutase, mitochondrial	MUT	1,06339433	+	
ATP-binding cassette sub-family D member 3	ABCD3	1,06370862	+	
Structural maintenance of chromosomes protein 4	SMC4	1,06396378	+	
Alcohol dehydrogenase [NADP(+)]	AKR1A1	1,06649611	+	
BTB/POZ domain-containing protein KCTD12	KCTD12;Kctd12	1,06699498	+	
DNA polymerase delta catalytic subunit	POLD1	1,06902334	+	
Band 4.1-like protein 2	EPB41L2	1,07153087	+	
26S protease regulatory subunit 10B	PSMC6	1,07276196	+	
Regulation of nuclear pre-mRNA domain-containing protein 1B	RPRD1B	1,07551363	+	
Cyclin-dependent kinase 1	CDK1	1,08203782	+	
Synapsin-1	SYN1	1,08556345	+	
Ornithine aminotransferase, mitochondrial;Ornithine aminotransferase, hepatic form;Ornithine aminotransferase, renal form	OAT	1,08617592	+	
28S ribosomal protein S22, mitochondrial	MRPS22	1,08682378	+	
tRNA-splicing ligase RtcB homolog	RTCB;rtcb	1,08863449	+	
Succinate dehydrogenase [ubiquinone] flavoprotein subunit, mitochondrial	SDHA	1,08875423	+	
SUMO-activating enzyme subunit 1;SUMO-activating enzyme subunit 1, N-terminally processed	SAE1	1,08953815	+	
3-mercaptopyruvate sulfurtransferase	MPST	1,09141858	+	
cAMP-dependent protein kinase catalytic subunit alpha	PRKACA	1,09949705	+	

Inosine-5-monophosphate dehydrogenase 2	IMPDH2	1,10493978	+	
Glucosamine-6-phosphate isomerase 1	GNPDA1	1,10534392	+	
Endoplasmic reticulum resident protein 44	ERP44	1,1056508	+	
ER membrane protein complex subunit 1	EMC1	1,10666614	+	
Delta-1-pyrroline-5-carboxylate synthase;Glutamate 5-kinase;Gamma-glutamyl phosphate reductase	ALDH18A1	1,10695733	+	
26S proteasome non-ATPase regulatory subunit 1	PSMD1	1,10745769	+	
Replication factor C subunit 4	RFC4	1,1082628	+	
Glutathione S-transferase omega-1	GSTO1	1,10830943	+	
Cytoplasmic dynein 1 light intermediate chain 1	DYNC1LI1	1,10837788	+	
NADH-ubiquinone oxidoreductase 75 kDa subunit, mitochondrial	NDUFS1	1,10967467	+	
Endophilin-A2	SH3GL1	1,11236042	+	
Elongation factor 1-delta	EEF1D	1,11504322	+	
Calpain-2 catalytic subunit	CAPN2	1,11789682	+	
Cytosol aminopeptidase	LAP3	1,11820115	+	
Probable ATP-dependent RNA helicase DDX46	DDX46	1,11878671	+	
Copine-3	CPNE3	1,12190289	+	
Nucleolar transcription factor 1	UBTF	1,12599924	+	
GTPase NRas	NRAS	1,13142776	+	
60S ribosomal protein L19	RPL19	1,13175625	+	
Ras-related protein Rab-21	RAB21	1,13661808	+	
Polyadenylate-binding protein 4	PABPC4	1,13796128	+	
DNA replication licensing factor MCM4	MCM4	1,14170096	+	
Low molecular weight phosphotyrosine protein phosphatase	ACP1	1,14384058	+	
Protein RER1	RER1	1,14668761	+	
Nuclear cap-binding protein subunit 1	NCBP1	1,14675395	+	

Sorting and assembly machinery component 50 homolog	SAMM50	1,14759827	+	
Serine/threonine-protein kinase 26	STK26	1,14860047	+	
Aminopeptidase B	RNPEP	1,15134536	+	
Diablo homolog, mitochondrial	DIABLO	1,15148841	+	
Cleft lip and palate transmembrane protein 1	CLPTM1	1,15618854	+	
Bleomycin hydrolase	BLMH	1,15647719	+	
RNA-binding protein with serine-rich domain 1	RNPS1	1,15653102	+	
Coronin-1A	CORO1A;Coro1a	1,15791215	+	
Sialic acid synthase	NANS	1,15846146	+	
DNA replication licensing factor MCM2	MCM2;Mcm2	1,1607009	+	
Transaldolase	TALDO1	1,16363313	+	
Synaptotagmin-1	Syt1;SYT1	1,16573736	+	
Eukaryotic translation initiation factor 5B	EIF5B	1,16850323	+	
Glycine cleavage system H protein, mitochondrial	GCSH	1,16958915	+	
Vacuolar protein sorting-associated protein 29	VPS29;vps29	1,17153422	+	
Exportin-T	XPOT	1,17379231	+	
Stomatin-like protein 2, mitochondrial	STOML2	1,17472013	+	
Nck-associated protein 1	NCKAP1;nckap1	1,17568694	+	
Chloride intracellular channel protein 4	CLIC4	1,17719523	+	
26S protease regulatory subunit 8	PSMC5	1,17812665	+	
Importin subunit alpha-1	KPNA2	1,1813221	+	
DNA (cytosine-5)-methyltransferase 1	DNMT1	1,18229421	+	
Calcium-binding mitochondrial carrier protein SCaMC-1	SLC25A24	1,1828732	+	
Proteasome activator complex subunit 3	PSME3	1,18618562	+	
Serine/threonine-protein phosphatase PGAM5, mitochondrial	PGAM5	1,18735949	+	
C-terminal-binding protein 1	CTBP1	1,18810251	+	

Ubiquitin-like modifier-activating enzyme 6	UBA6	1,18917169	+	
Mitochondrial carrier homolog 2	MTCH2	1,18970256	+	
Ribonucleoside-diphosphate reductase subunit M2	RRM2	1,19053099	+	
S-phase kinase-associated protein 1	SKP1	1,19096947	+	
Coatomer subunit gamma-1	COPG1	1,19160737	+	
Exportin-5	XPO5	1,19247754	+	
Histidine triad nucleotide-binding protein 1	HINT1	1,19640308	+	
Transforming protein RhoA	Rhoa;RHOA;rhoab	1,20195495	+	
Structural maintenance of chromosomes protein 2	SMC2	1,20228174	+	
LETM1 and EF-hand domain-containing protein 1, mitochondrial	LETM1	1,20574273	+	
Double-stranded RNA-binding protein Staufen homolog 1	STAU1	1,21546872	+	
SWI/SNF complex subunit SMARCC1	SMARCC1;Smarcc1	1,21738921	+	
Macrophage migration inhibitory factor	MIF;Mif	1,21811401	+	
Trifunctional enzyme subunit beta, mitochondrial;3-ketoacyl-CoA thiolase	HADHB	1,21991603	+	
E3 ubiquitin-protein ligase UBR4	UBR4	1,22438007	+	
Mitochondrial import receptor subunit TOM40 homolog	TOMM40	1,22960154	+	
Structural maintenance of chromosomes flexible hinge domain-containing protein 1	SMCHD1	1,23066839	+	
SUMO-conjugating enzyme UBC9	Ube2i;UBE2I	1,23218918	+	
Eukaryotic translation initiation factor 5	EIF5	1,2381293	+	
1-phosphatidylinositol 4,5-bisphosphate phosphodiesterase gamma-1	PLCG1	1,24022484	+	
Methylsterol monooxygenase 1	MSMO1	1,24659877	+	
Tryptophan--tRNA ligase, cytoplasmic;T1-TrpRS;T2-TrpRS	WARS	1,24817318	+	

Microtubule-associated protein RP/EB family member 2	MAPRE2	1,24869453	+	
Symplekin	SYMPK	1,24962786	+	
Glyoxylate reductase/hydroxypyruvate reductase	GRHPR	1,24965096	+	
4-trimethylaminobutyraldehyde dehydrogenase	ALDH9A1	1,25107638	+	
N-alpha-acetyltransferase 15, NatA auxiliary subunit	NAA15	1,26060253	+	
tRNA (cytosine(34)-C(5))-methyltransferase	NSUN2	1,26419301	+	
Ribose-phosphate pyrophosphokinase 1	PRPS1	1,26651658	+	
Eukaryotic translation initiation factor 5A-1;Eukaryotic translation initiation factor 5A-1-like;Eukaryotic translation initiation factor 5A-2	EIF5A;EIF5AL1;EIF5A2	1,26715575	+	
Gamma-glutamyl hydrolase	GGH	1,26757537	+	
Rab GTPase-activating protein 1	RABGAP1	1,26782735	+	
CUGBP Elav-like family member 1	CELF1;Celf1	1,27082994	+	
Nuclear pore complex protein Nup93	NUP93	1,27744166	+	
Tubulin polymerization-promoting protein family member 3	TPPP3	1,27820799	+	
Importin subunit alpha-4	Kpna3;KPNA3	1,27962981	+	
Double-stranded RNA-binding protein Staufen homolog 2	STAU2	1,28062312	+	
Small nuclear ribonucleoprotein Sm D1	SNRPD1	1,28078588	+	
26S proteasome non-ATPase regulatory subunit 7	PSMD7	1,29013634	+	
SH3 and PX domain-containing protein 2B	SH3PXD2B	1,29430347	+	
EH domain-containing protein 1	EHD1	1,29486889	+	
Creatine kinase U-type, mitochondrial	CKMT1A	1,29677412	+	
AP-1 complex subunit beta-1	AP1B1;Ap1b1	1,29709562	+	
Echinoderm microtubule-associated protein-like 4	EML4	1,29756249	+	

Transportin-1	TNPO1	1,2988063	+	
Junction plakoglobin	JUP	1,30739339	+	
Polyadenylate-binding protein-interacting protein 1	PAIP1	1,3093124	+	
Spermidine synthase	SRM	1,31046973	+	
Superoxide dismutase [Mn], mitochondrial	SOD2	1,31601312	+	
Calponin-2	CNN2	1,32280413	+	
Proline-, glutamic acid- and leucine-rich protein 1	PELP1	1,32325384	+	
Nodal modulator 2;Nodal modulator 3;Nodal modulator 1	NOMO2;NOMO3;NON	1,33712133	+	
DNA replication licensing factor MCM5	MCM5	1,34007666	+	
Fructose-bisphosphate aldolase C	ALDOC	1,34261025	+	
Phosphomevalonate kinase	PMVK	1,3451182	+	
Leucine--tRNA ligase, cytoplasmic	LARS	1,3463548	+	
GMP synthase [glutamine-hydrolyzing]	GMPS	1,34731123	+	
Vacuolar protein sorting-associated protein 26A	VPS26A	1,34943602	+	
Acylglycerol kinase, mitochondrial	AGK	1,35339419	+	
Sterol-4-alpha-carboxylate 3-dehydrogenase, decarboxylating	NSDHL	1,35563893	+	
Replication factor C subunit 2	RFC2;Rfc2	1,36727333	+	
Phosphoribosyl pyrophosphate synthase-associated protein 2	PRPSAP2	1,37010574	+	
Translationally-controlled tumor protein	TPT1	1,37118318	+	
L-lactate dehydrogenase A chain	LDHA	1,37382317	+	
Serotransferrin	TF	1,37452168	+	
rRNA 2-O-methyltransferase fibrillarin	FBL	1,37890095	+	
Cilia- and flagella-associated protein 20	CFAP20	1,38244353	+	
Asparagine synthetase [glutamine-hydrolyzing]	ASNS	1,38304392	+	
Myelin expression factor 2	MYEF2	1,38640277	+	

Ubiquitin carboxyl-terminal hydrolase isozyme L5	UCHL5	1,3929437	+	
NAD(P) transhydrogenase, mitochondrial	NNT	1,39506065	+	
Serine/threonine-protein kinase VRK1	VRK1	1,3966287	+	
BolA-like protein 2	BOLA2	1,39900398	+	
Serine/threonine-protein phosphatase PP1-alpha catalytic subunit	PPP1CA	1,40087403	+	
40S ribosomal protein S15a	RPS15A	1,40176688	+	
DNA replication licensing factor MCM3	MCM3	1,4035812	+	
Programmed cell death 6-interacting protein	PDCD6IP	1,40365707	+	
Aldose reductase	AKR1B1	1,40620316	+	
Protein ERGIC-53	LMAN1	1,40865495	+	
Amidophosphoribosyltransferase	PPAT	1,40898344	+	
AP-3 complex subunit beta-1	AP3B1	1,40919198	+	
V-type proton ATPase 116 kDa subunit a isoform 1	ATP6V0A1	1,4100469	+	
Dihydrofolate reductase	DHFR	1,41320165	+	
60S ribosomal protein L10	RPL10	1,42001449	+	
Casein kinase II subunit alpha;Casein kinase II subunit alpha 3	Csnk2a1;CSNK2A1;CSI	1,42455716	+	
Rabankyrin-5	ANKFY1	1,430201	+	
Lactoylglutathione lyase	GLO1	1,44422616	+	
ATP-dependent 6-phosphofructokinase, liver type	PFKL	1,44787788	+	
DnaJ homolog subfamily B member 11	DNAJB11	1,44850201	+	
Cytochrome b5 type B	CYB5B	1,4577891	+	
ATP-binding cassette sub-family E member 1	ABCE1	1,46688165	+	
Neural cell adhesion molecule L1	L1CAM	1,46708934	+	
Beta-soluble NSF attachment protein	NAPB	1,46821361	+	
Superkiller viralicidic activity 2-like 2	SKIV2L2	1,47076183	+	
Histone-arginine methyltransferase CARM1	CARM1	1,47321447	+	

AP-3 complex subunit mu-1	AP3M1	1,47604307	+	
Signal transducer and activator of transcription 1-alpha/beta	STAT1	1,4764542	+	
Copine-1	CPNE1	1,48263359	+	
Single-stranded DNA-binding protein, mitochondrial	SSBP1	1,48416011	+	
Thioredoxin reductase 1, cytoplasmic	TXNRD1	1,48630291	+	
Lethal(2) giant larvae protein homolog 1	LLGL1;Llgl1	1,48706245	+	
Glutamine--fructose-6-phosphate aminotransferase [isomerizing] 1	GFPT1	1,4924433	+	
Plastin-3	PLS3	1,49344381	+	
Ankyrin-2	ANK2	1,49850718	+	
3-ketoacyl-CoA thiolase, mitochondrial	ACAA2	1,49996482	+	
Dolichyl-diphosphooligosaccharide--protein glycosyltransferase subunit DAD1	Dad1;DAD1	1,50628026	+	
Cysteine and glycine-rich protein 2	CSRP2	1,51664988	+	
Ribonucleoside-diphosphate reductase large subunit	RRM1	1,51934963	+	
Ras-related protein Rab-2B	RAB2B	1,52369372	+	
Small nuclear ribonucleoprotein Sm D3	SNRPD3	1,52862612	+	
Vesicle-trafficking protein SEC22b	SEC22B	1,53125276	+	
Ubiquitin carboxyl-terminal hydrolase 10	USP10	1,53869883	+	
Eukaryotic peptide chain release factor subunit 1	ETF1	1,54195086	+	
Ras GTPase-activating-like protein IQGAP1	IQGAP1	1,54405997	+	
Aminoacyl tRNA synthase complex-interacting multifunctional protein 1;Endothelial monocyte-activating polypeptide 2	AIMP1	1,55432426	+	
ADP-ribosylation factor 1;ADP-ribosylation factor 3	Arf1;ARF1;ARF3	1,562301	+	

ATPase family AAA domain-containing protein 1;ATPase family AAA domain-containing protein 1-B	ATAD1;atad1b	1,56366009	+	
Condensin complex subunit 1	NCAPD2	1,57342254	+	
Putative ATP-dependent RNA helicase DHX30	DHX30	1,57458687	+	
DnaJ homolog subfamily A member 1	DNAJA1;Dnaja1	1,57503446	+	
Transcription elongation factor B polypeptide 1	TCEB1	1,58280585	+	
2-oxoglutarate dehydrogenase, mitochondrial	OGDH	1,59054989	+	
RNA-binding protein 4	RBM4	1,59804175	+	
CDGSH iron-sulfur domain-containing protein 2	CISD2;Cisd2	1,60473484	+	
COP9 signalosome complex subunit 2	COPS2	1,61503855	+	
Four and a half LIM domains protein 1	FHL1;Fhl1	1,61602105	+	
26S proteasome non-ATPase regulatory subunit 12	PSMD12	1,61712053	+	
Eukaryotic translation initiation factor 3 subunit I	EIF3I	1,63669247	+	
NHP2-like protein 1;NHP2-like protein 1, N-terminally processed	NHP2L1	1,64543682	+	
mRNA export factor	RAE1	1,64564217	+	
Cancer-related nucleoside-triphosphatase	NTPCR	1,65407817	+	
Serine/threonine-protein phosphatase 2A 56 kDa regulatory subunit epsilon isoform	PPP2R5E	1,65412649	+	
DNA replication licensing factor MCM7	MCM7	1,66373634	+	
60S ribosomal protein L27	RPL27	1,66913626	+	
26S proteasome non-ATPase regulatory subunit 5	PSMD5	1,67783546	+	
Acetyl-CoA acetyltransferase, cytosolic	ACAT2	1,68484137	+	
Solute carrier family 2, facilitated glucose transporter member 1	SLC2A1	1,7083274	+	

Perilipin-3	PLIN3	1,71646457	+	
Delta(24)-sterol reductase	DHCR24	1,73236232	+	
FAS-associated factor 2	FAF2	1,73505211	+	
Heat shock 70 kDa protein 4L	HSPA4L	1,7407928	+	
Glypican-4;Secreted glypican-4	GPC4	1,75487497	+	
Small nuclear ribonucleoprotein-associated proteins B and B	SNRPB	1,75680796	+	
Importin subunit alpha-5;Importin subunit alpha-5, N-terminally processed	KPNA1;Kpna1	1,76424514	+	
Myotrophin	MTPN	1,7719858	+	
Aminoacyl tRNA synthase complex-interacting multifunctional protein 2	AIMP2	1,78462558	+	
Thioredoxin	TXN	1,79231008	+	
Actin-related protein 2/3 complex subunit 4	Arpc4;ARPC4	1,79283778	+	
40S ribosomal protein S27-like	RPS27L	1,79476293	+	
Eukaryotic initiation factor 4A-II;Eukaryotic initiation factor 4A-II, N-terminally processed	EIF4A2	1,8018411	+	
S-adenosylmethionine synthase isoform type-2	MAT2A	1,80378787	+	
Leucine-rich repeat-containing protein 40	LRRC40	1,80677817	+	
LIM and SH3 domain protein 1	LASP1	1,80794885	+	
Elongation factor 1-alpha 2	EEF1A2;Eef1a2	1,80938085	+	
Small glutamine-rich tetratricopeptide repeat-containing protein alpha	SGTA	1,82048522	+	
D-3-phosphoglycerate dehydrogenase	PHGDH	1,82128949	+	
Actin-like protein 6A	ACTL6A;Actl6a	1,82179472	+	
Histone-binding protein RBBP7	RBBP7	1,86049122	+	
Ran-specific GTPase-activating protein	RANBP1;Ranbp1	1,8705438	+	
BUB3-interacting and GLEBS motif-containing protein ZNF207	ZNF207	1,87806087	+	
Unconventional myosin-Ib	MYO1B	1,88188214	+	

Pyruvate dehydrogenase E1 component subunit alpha, somatic form, mitochondrial	PDHA1	1,88665432	+	
Ras-related protein Rab-6B	RAB6B	1,91266653	+	
Mitochondrial glutamate carrier 1;Mitochondrial glutamate carrier 2	SLC25A22;SLC25A18	1,91282908	+	
Gamma-synuclein	SNCG	1,91344664	+	
Tyrosine-protein phosphatase non-receptor type 1	PTPN1	1,91481972	+	
Ras-related protein Rab-10	RAB10	1,94398859	+	
Translational activator GCN1	GCN1L1	1,94721307	+	
DNA mismatch repair protein Msh6	MSH6	1,95109749	+	
Activator of 90 kDa heat shock protein ATPase homolog 1	AHSA1	1,95730146	+	
Aldehyde dehydrogenase family 16 member A1	ALDH16A1	1,98171404	+	
Phosphoribosylformylglycinamidine synthase	PFAS	1,99841309	+	
Thymidylate synthase	TYMS	1,99952147	+	
Medium-chain specific acyl-CoA dehydrogenase, mitochondrial	ACADM	2,0000568	+	
Peptidyl-prolyl cis-trans isomerase-like 1	PPIL1	2,01126713	+	
Inorganic pyrophosphatase	PPA1	2,02653122	+	
Protein FAM98B	FAM98B	2,05904028	+	
Phospholipid hydroperoxide glutathione peroxidase, mitochondrial	GPX4	2,06965658	+	
Protein NipSnap homolog 1	NIPSNAP1;Nipsnap1	2,08286815	+	
Histone deacetylase 2	HDAC2;Hdac2	2,13791275	+	
Cellular retinoic acid-binding protein 2	CRABP2	2,14412202	+	
Eukaryotic translation initiation factor 2 subunit 2	EIF2S2	2,14471118	+	
26S proteasome non-ATPase regulatory subunit 6	PSMD6	2,15996827	+	
Adenylate kinase isoenzyme 1	AK1	2,16386965	+	

Dynein light chain 2, cytoplasmic	DYNLL2	2,23186874	+	
GTP-binding protein SAR1a	SAR1A	2,24796465	+	
Tricarboxylate transport protein, mitochondrial	SLC25A1	2,28178279	+	
Ubiquitin-conjugating enzyme E2 N;Ubiquitin-conjugating enzyme E2 35;Ubiquitin-conjugating enzyme E2 36;Putative ubiquitin-conjugating enzyme E2 N-like	UBE2N;Ube2n;UBC35	2,29580328	+	
Signal recognition particle 9 kDa protein	SRP9	2,31755235	+	
DNA topoisomerase 2-alpha	TOP2A	2,34728707	+	
Saccharopine dehydrogenase-like oxidoreductase	SCCPDH	2,37124464	+	
Peroxiredoxin-4	PRDX4;Prdx4	2,38741154	+	
60S ribosomal protein L26;60S ribosomal protein L26-like 1	RPL26;RPL26L1	2,44196616	+	
Thymidylate kinase	DTYMK	2,60647668	+	
L-xylulose reductase	DCXR	2,62080744	+	
Tubulin alpha-1B chain;Tubulin alpha-4A chain	TUBA1B;TUBA4A	2,72059165	+	

Supplementary Table 3. List of the 118 dysregulated proteins in p.A53T neurons that were restored upon treatment with BX795

**Gene Name**	**Protein Name**	**Biological Process**	**-Log ANOVA p v**	**ANOVA q-value**
SH3GL1	Endophilin-A2	Cell Membrane	256.133	0,011476
MIF	Macrophage migration inhibitory facto	Cytokine	180.931	0,0363767
ACTR2	Actin-related protein 2	Cytoskeleton	194.818	0,030058
CAPZB	F-actin-capping protein subunit beta	Cytoskeleton	46.958	0,000648649
DYNLL2	Dynein light chain 2, cytoplasmic	Cytoskeleton	278.624	0,00791795
JUP	Junction plakoglobin	Cytoskeleton	184.446	0,0338618
MARCKSL1	MARCKS-related protein	Cytoskeleton	182.146	0,0355364
SNCG	Gamma-synuclein	Cytoskeleton	369.107	0,0025
TUBB	Tubulin beta chain	Cytoskeleton	328.033	0,004
HAT1	Histone acetyltransferase type B cataly	DNA Organization	193.087	0,0303367
ACO2	Aconitate hydratase, mitochondrial	Metabolism	239.553	0,0147068
ACP1	Low molecular weight phosphotyrosine	Metabolism	223.884	0,0187195
ALDOC	Fructose-bisphosphate aldolase C	Metabolism	19.283	0,0305445
DCXR	L-xylulose reductase	Metabolism	450.627	0,000888889
DTYMK	Thymidylate kinase	Metabolism	429.648	0,00128302
GPX4	Phospholipid hydroperoxide glutathion	Metabolism	313.215	0,00468571
MSMO1	Methylsterol monooxygenase 1	Metabolism	23.571	0,0159706
NANS	Sialic acid synthase	Metabolism	197.987	0,0284313
OGDH	2-oxoglutarate dehydrogenase, mitoch	Metabolism	189.588	0,0314673
TSTA3	GDP-L-fucose synthase	Metabolism	471.268	0,000666667
ALDH5A1	Succinate-semialdehyde dehydrogenas	Neuronal	281.456	0,00780645
ATXN10	Ataxin-10	Neuronal	36.278	0,00266667
INA	Alpha-internexin	Neuronal	261.725	0,0103014
NIPSNAP1	Protein NipSnap homolog 1	Neuronal	201.319	0,0272045
PAFAH1B1	Platelet-activating factor acetylhydrola	Neuronal	416.529	0,00144262
SYN1	Synapsin-1	Neuronal	282.487	0,00773913
HIST1H1E	Histone H1.4	Nuclear Assembly	462.922	0,000682927
TMPO	Lamina-associated polypeptide 2, isofo	Nuclear Assembly	273.826	0,00829268
ACADM	Medium-chain specific acyl-CoA dehyd	Oxidative Stress	361.925	0,00273684
CKMT1A	Creatine kinase U-type, mitochondrial	Oxidative Stress	169.957	0,0433538
GOT2	Aspartate aminotransferase, mitochon	Oxidative Stress	195.739	0,0293862

GPX1	Glutathione peroxidase 1	Oxidative Stress	256.414	0,0115088
MDH2	Malate dehydrogenase, mitochondrial	Oxidative Stress	325.647	0,00410256
MTCH2	Mitochondrial carrier homolog 2	Oxidative Stress	2.402	0,0146818
PDHA1	Pyruvate dehydrogenase E1 componen	Oxidative Stress	222.971	0,0189673
STOML2	Stomatin-like protein 2, mitochondrial	Oxidative Stress	313.378	0,00475362
TOMM70A	Mitochondrial import receptor subunit	Oxidative Stress	181.295	0,0362534
ATP1B1	Sodium/potassium-transporting ATPas	Plasma Membrane	192.654	0,0305888
ANKFY1	Rabankyrin-5	Protein Modification and Transpo	23.323	0,0163
AP3M1	AP-3 complex subunit mu-1	Protein Modification and Transpo	178.641	0,0375263
ATP6V0D1	V-type proton ATPase subunit d 1	Protein Modification and Transpo	186.993	0,0326792
CCT8	T-complex protein 1 subunit theta	Protein Modification and Transpo	449.206	0,000956522
CKAP4	Cytoskeleton-associated protein 4	Protein Modification and Transpo	271.468	0,00867308
DAD1	Dolichyl-diphosphooligosaccharide--pr	Protein Modification and Transpo	270.969	0,00872381
GDI2	Rab GDP dissociation inhibitor beta	Protein Modification and Transpo	194.495	0,0300524
LAP3	Cytosol aminopeptidase	Protein Modification and Transpo	19.891	0,0281967
NAPB	Beta-soluble NSF attachment protein	Protein Modification and Transpo	263.779	0,00978802
OGT	UDP-N-acetylglucosamine--peptide N-a	Protein Modification and Transpo	216.824	0,0208777
PACSIN2	Protein kinase C and casein kinase subs	Protein Modification and Transpo	286.533	0,00715556
PLIN3	Perilipin-3	Protein Modification and Transpo	167.058	0,0455219
RAB2B	Ras-related protein Rab-2B	Protein Modification and Transpo	377.583	0,00245783
SAR1A	GTP-binding protein SAR1a	Protein Modification and Transpo	227.985	0,0173559
SEC22B	Vesicle-trafficking protein SEC22b	Protein Modification and Transpo	189.207	0,0317391
SRP9	Signal recognition particle 9 kDa protei	Protein Modification and Transpo	357.866	0,00273469
YKT6	Synaptobrevin homolog YKT6	Protein Modification and Transpo	201.411	0,0271685
AIMP2	Aminoacyl tRNA synthase complex-inte	Protein Synthesis	540.207	0,00032
EEF1D	Elongation factor 1-delta	Protein Synthesis	24.002	0,0146566
EIF2B4	Translation initiation factor eIF-2B sub	Protein Synthesis	277.038	0,00814141
EIF4G2	Eukaryotic translation initiation factor	Protein Synthesis	325.565	0,00403361
FAM98B	Protein FAM98B	Protein Synthesis	302.174	0,00571795
GTPBP4	Nucleolar GTP-binding protein 1	Protein Synthesis	178.873	0,0375154
KARS	Lysine--tRNA ligase	Protein Synthesis	359.204	0,00272165
MAT2A	S-adenosylmethionine synthase isofor	Protein Synthesis	350.993	0,00290196
PHGDH	D-3-phosphoglycerate dehydrogenase	Protein Synthesis	106.486	0

PPA1	Inorganic pyrophosphatase	Protein Synthesis	203.525	0,0261486
PRMT1	Protein arginine N-methyltransferase 1	Protein Synthesis	558.198	0,000190476
RARS	Arginine--tRNA ligase, cytoplasmic	Protein Synthesis	34.511	0,00316981
RPL12	60S ribosomal protein L12	Protein Synthesis	236.748	0,0156444
RPL31	60S ribosomal protein L31	Protein Synthesis	241.579	0,0145385
RPS3	40S ribosomal protein S3	Protein Synthesis	321.674	0,0043252
RPS6	40S ribosomal protein S6	Protein Synthesis	295.529	0,0064878
RTCB	tRNA-splicing ligase RtcB homolog	Protein Synthesis	347.921	0,00303846
VARS	Valine--tRNA ligase	Protein Synthesis	302.941	0,00571429
WARS	Tryptophan--tRNA ligase, cytoplasmic;T	Protein Synthesis	233.632	0,0162437
DEK	Protein DEK	RNA Metabolism	19.105	0,0308276
HINT1	Histidine triad nucleotide-binding prote	RNA Metabolism	417.925	0,00135593
HNRNPUL1	Heterogeneous nuclear ribonucleoprot	RNA Metabolism	213.047	0,0220917
MYEF2	Myelin expression factor 2	RNA Metabolism	178.281	0,0377856
NHP2L1	NHP2-like protein 1;NHP2-like protein	RNA Metabolism	395.001	0,00191781
NUP93	Nuclear pore complex protein Nup93	RNA Metabolism	290.302	0,00691954
PCBP1	Poly(rC)-binding protein 1	RNA Metabolism	617.692	0
PCBP2	Poly(rC)-binding protein 2	RNA Metabolism	242.141	0,0143938
RAE1	mRNA export factor	RNA Metabolism	231.102	0,0169645
RBM4	RNA-binding protein 4	RNA Metabolism	23.421	0,0162464
RRM2	Ribonucleoside-diphosphate reductase	RNA Metabolism	311.101	0,00472222
SKIV2L2	Superkiller viralicidic activity 2-like 2	RNA Metabolism	395.007	0,00194444
SNRPB	Small nuclear ribonucleoprotein-associ	RNA Metabolism	19.464	0,0300842
UBTF	Nucleolar transcription factor 1	RNA Metabolism	252.596	0,0123729
YLPM1	YLP motif-containing protein 1	RNA Metabolism	347.249	0,00304762
ZNF207	BUB3-interacting and GLEBS motif-con	RNA Metabolism	280.434	0,00785263
API5	Apoptosis inhibitor 5	Signal Transduction	192.253	0,03041
BOLA2	BolA-like protein 2	Signal Transduction	243.992	0,0139765
CRABP2	Cellular retinoic acid-binding protein 2	Signal Transduction	652.555	0
CSK	Tyrosine-protein kinase CSK	Signal Transduction	302.647	0,00570323
MTPN	Myotrophin	Signal Transduction	536.379	0,000307692
STAT1	Signal transducer and activator of trans	Signal Transduction	250.904	0,0127197
ZYX	Zyxin	Signal Transduction	323.528	0,00406557

ATG4B	Cysteine protease ATG4B	Stress Response	30.765	0,00512752
CUL1	Cullin-1	Stress Response	205.844	0,0253684
DNAJA1	DnaJ homolog subfamily A member 1	Stress Response	264.155	0,00980465
DNAJB11	DnaJ homolog subfamily B member 11	Stress Response	234.026	0,0161727
DNM2	Dynamin-2	Stress Response	185.961	0,0332459
GCN1L1	Translational activator GCN1	Stress Response	57.176	0
HSPA4	Heat shock 70 kDa protein 4	Stress Response	325.383	0,004
OTUB1	Ubiquitin thioesterase OTUB1	Stress Response	351.675	0,00289109
PDCD6IP	Programmed cell death 6-interacting p	Stress Response	300.728	0,00585987
PSMA3	Proteasome subunit alpha type-3	Stress Response	318.164	0,00443077
PSMD12	26S proteasome non-ATPase regulator	Stress Response	269.053	0,00904265
PSME3	Proteasome activator complex subunit	Stress Response	331.891	0,00378947
PTPN1	Tyrosine-protein phosphatase non-rec	Stress Response	349.068	0,00291262
SGTA	Small glutamine-rich tetratricopeptide	Stress Response	191.864	0,0304814
STIP1	Stress-induced-phosphoprotein 1	Stress Response	303.961	0,00565789
TCP1	T-complex protein 1 subunit alpha	Stress Response	480.438	0,000685714
UBA6	Ubiquitin-like modifier-activating enzy	Stress Response	226.136	0,0179532
UCHL1	Ubiquitin carboxyl-terminal hydrolase i	Stress Response	555.947	0,000181818
VCP	Transitional endoplasmic reticulum ATI	Stress Response	263.882	0,00983333
VPS35	Vacuolar protein sorting-associated pro	Stress Response	303.784	0,00564706
TPD52L2	Tumor protein D54	Unknown	19.239	0,0305592

pplementary Table 4. GO analysis for cellular compartment between pA53T and control neuro

**Cellular Compartment**	**No of genes**	**P-Value**	**Bonferroni**
extracellular exosome	299	1,6E-75	1,0E-72
membrane	204	2,8E-38	1,7E-35
nucleoplasm	230	7,9E-36	4,8E-33
cytoplasm	334	2,2E-32	1,3E-29
mitochondrion	118	1,6E-19	9,5E-17
nucleus	292	6,3E-15	3,9E-12
nuclear pore	22	6,7E-14	4,1E-11
intracellular ribonucleoprotein complex	29	7,5E-14	4,6E-11
nucleosome	23	2,4E-12	1,4E-9
nuclear chromosome, telomeric region	26	8,1E-12	4,9E-9
nuclear nucleosome	16	2,1E-11	1,3E-8
nuclear envelope	28	2,5E-11	1,5E-8
proteasome complex	18	2,8E-11	1,7E-8
focal adhesion	43	4,4E-10	2,7E-7
eukaryotic translation initiation factor 3 complex	10	2,1E-9	1,3E-6
proteasome accessory complex	9	5,6E-8	3,4E-5
chaperonin-containing T-complex	7	1,8E-7	1,1E-4
nuclear membrane	27	3,3E-7	2,0E-4
cell body	14	3,6E-7	2,2E-4
proteasome regulatory particle	7	9,3E-7	5,7E-4
eukaryotic translation initiation factor 3 complex	6	1,3E-6	7,8E-4
axon cytoplasm	10	3,40E-06	7,3E-5

Supplementary Table 5. Primers used in the current study

Gene name	Application	Forward	Reverse
TH	RT-PCR	TGTCTGAGGAGCCTGAGATTCG	GCTTGTCCTTGGCGTCACTG
Nurr1	RT-PCR	TCGACATTTCTGCCTTCTCCTG	GGTTCCTTGAGCCCGTGTCT
AADC	RT-PCR	TGCGAGCAGAGAGGGAGTAG	TGAGTTCCATGAAGGCAGGATG

Supplementary Table 6. Primary antibodies used in the current study

Name	Host	Dilution	Vendor	Catalog#
Anti-GAPDH	Mouse	1/1000	Santa Cruz Biotechnology	sc-365062
Anti-beta actin	Mouse	1/5000	Abcam	ab8227
Anti-MAP2	Mouse	1/200	Merck-Millipore	MAB3418
Anti-NESTIN	Rabbit	1/200	Merck-Millipore	ABD69
Anti-α-Synuclein (αSyn)	Mouse	1/500	BD Biosciences	610787
Anti-phosphorylated α-Synuclein (Ser129)	Mouse	1/10000	WAKO	015-25191
Anti-TH	Rabbit	1/500	Merck-Millipore	AB152
Anti-VGLUT1	Mouse	1/1000	Merck-Millipore	MAB5502
Anti-TUJ1	Mouse	1/1000	Biolegend	801202
Anti-PAX6	Mouse	1/100	DSHB	AB 528427
Anti-ki67	Rabbit	1/400	Abcam	ab15580
Anti-Phospho-S6 Ribosomal Protein (Ser235/236)	Rabbit	1/1000	Cell Signalling	4858
Anti-S6 Ribosomal Protein (5G10)	Rabbit	1/1000	Cell Signalling	2217
Anti-Phospho-mTOR (Ser2448) (D9C2)	Rabbit	1/1000	Cell Signalling	5536
Anti- mTOR (7C10)	Rabbit	1/1000	Cell Signalling	2983
Anti-Phospho-PRAS40 (Thr246) (C77D7)	Rabbit	1/1000	Cell Signalling	2997
Anti-PRAS40 (D23C7)	Rabbit	1/1000	Cell Signalling	2691
Anti-TBK1/NAK	Rabbit	1/1000	Cell Signalling	3013
Anti-Phospho-TBK1/NAK (Ser172) (D52C2)	Rabbit	1/1000	Cell Signalling	5483
Anti-Phospho-PDK1 (Ser241)	Rabbit	1/1000	Cell Signalling	3061
Anti-PDK1 (D37A7)	Rabbit	1/1000	Cell Signalling	5662
