# Supplementary material for: High content screening and proteomic analysis identify a kinase inhibitor that rescues pathological phenotypes in a patient-derived model of Parkinson’s disease
Source: NPJ Parkinsons Dis. 2022 Feb 11;8:15. doi: 10.1038/s41531-022-00278-y (PMC8837749; doi:10.1038/s41531-022-00278-y)
Supplement: Supplementary file 2 — Reporting Summary [file 41531_2022_278_MOESM2_ESM.pdf]

nature portfolio

Corresponding author(s): Rebecca Matsas

Last updated by author(s): Sep 7, 2021

Reporting Summary

Nature Portfolio wishes to improve the reproducibility of the work that we publish. This form provides structure for consistency and transparency in reporting. For further information on Nature Portfolio policies, see our Editorial Policies and the Editorial Policy Checklist.

Statistics

For all statistical analyses, confirm that the following items are present in the figure legend, table legend, main text, or Methods section.

n/a	Confirmed	
☐	☒	The exact sample size (n) for each experimental group/condition, given as a discrete number and unit of measurement
☐	☒	A statement on whether measurements were taken from distinct samples or whether the same sample was measured repeatedly
☐	☒	The statistical test(s) used AND whether they are one- or two-sided<br>*Only common tests should be described solely by name; describe more complex techniques in the Methods section.*
☐	☒	A description of all covariates tested
☐	☒	A description of any assumptions or corrections, such as tests of normality and adjustment for multiple comparisons
☐	☒	A full description of the statistical parameters including central tendency (e.g. means) or other basic estimates (e.g. regression coefficient) AND variation (e.g. standard deviation) or associated estimates of uncertainty (e.g. confidence intervals)
☐	☒	For null hypothesis testing, the test statistic (e.g. F, t, r) with confidence intervals, effect sizes, degrees of freedom and P value noted<br>*Give P values as exact values whenever suitable.*
☒	☐	For Bayesian analysis, information on the choice of priors and Markov chain Monte Carlo settings
☐	☒	For hierarchical and complex designs, identification of the appropriate level for tests and full reporting of outcomes
☒	☐	Estimates of effect sizes (e.g. Cohen's d, Pearson's r), indicating how they were calculated

Our web collection on statistics for biologists contains articles on many of the points above.

Software and code

Policy information about availability of computer code

Data collection	For High-Content Screening images were captured by automated confocal microscopy (Opera High-Content Screening System, Perkin Elmer, Hamburg, Germany). Confocal Images were captured using Leica TCS SP8 confocal microscope (LEICA Microsystems) . The raw mass spectral files were processed using MaxQuant software (version 1.6.9.0) with default parameters for protein identification and quantification.
Data analysis	Statistical analysis for proteomics data was performed using Perseus (1.6.6.0). Enrichment analysis for biological processes, molecular function and cellular compartment was performed using DAVID functional annotation tools.The enrichment of proteins involved in signaling pathways was performed using the Reactome pathway database. Confocal images were analyzed using ImageJ software (NIH).Statistical analysis was performed using GraphPad Prism 6 software.

For manuscripts utilizing custom algorithms or software that are central to the research but not yet described in published literature, software must be made available to editors and reviewers. We strongly encourage code deposition in a community repository (e.g. GitHub). See the Nature Portfolio guidelines for submitting code & software for further information.

Data

Policy information about availability of data

All manuscripts must include a data availability statement. This statement should provide the following information, where applicable:

- Accession codes, unique identifiers, or web links for publicly available datasets
- A description of any restrictions on data availability
- For clinical datasets or third party data, please ensure that the statement adheres to our policy

The mass spectrometry proteomics data have been deposited to the ProteomeXchange Consortium via the PRIDE partner repository with the dataset identifier PXD019574.

Field-specific reporting

Please select the one below that is the best fit for your research. If you are not sure, read the appropriate sections before making your selection.

☒ Life sciences ☐ Behavioural & social sciences ☐ Ecological, evolutionary & environmental sciences

For a reference copy of the document with all sections, see nature.com/documents/nr-reporting-summary-flat.pdf

Life sciences study design

All studies must disclose on these points even when the disclosure is negative.

Sample size	Sample sizes were not predetermined, instead followed previous publications from our lab and others.
Data exclusions	No data was excluded.
Replication	All data was generated from multiple biological replicates. Additionally, the number of independent experiments is shown.
Randomization	All samples were assigned treatments randomly.
Blinding	All experiments and analysis were performed randomly.

Reporting for specific materials, systems and methods

We require information from authors about some types of materials, experimental systems and methods used in many studies. Here, indicate whether each material, system or method listed is relevant to your study. If you are not sure if a list item applies to your research, read the appropriate section before selecting a response.

Materials & experimental systems

n/a	Involved in the study	
☐	☒	Antibodies
☐	☒	Eukaryotic cell lines
☒	☐	Palaeontology and archaeology
☒	☐	Animals and other organisms
☒	☐	Human research participants
☒	☐	Clinical data
☒	☐	Dual use research of concern

Methods

n/a	Involved in the study	
☒	☐	ChIP-seq
☒	☐	Flow cytometry
☒	☐	MRI-based neuroimaging

Antibodies

Antibodies used

Name Host Dilution Vendor Catalog#
Anti-GAPDH Mouse 1/1000 Santa Cruz Biotechnology sc-365062
Anti-beta actin Mouse 1/5000 Abcam ab8227
Anti-MAP2 Mouse 1/200 Merck-Millipore MAB3418
Anti-NESTIN Rabbit 1/200 Merck-Millipore ABD69
Anti-α-Synuclein (αSyn) Mouse 1/500 BD Biosciences 610787
Anti-phosphorylated α-Synuclein (Ser129) Mouse 1/10000 WAKO 015-25191
Anti-TH Rabbit 1/500 Merck-Millipore AB152
Anti-VGLUT1 Mouse 1/1000 Merck-Millipore MAB5502
Anti-TUJ1 Mouse 1/1000 Biolegend 801202
Anti-PAX6 Mouse 1/100 DSHB AB 528427
Anti-ki67 Rabbit 1/400 Abcam ab15580
Anti-Phospho-S6 Ribosomal Protein (Ser235/236) Rabbit 1/1000 Cell Signalling 4858
Anti-S6 Ribosomal Protein (5G10) Rabbit 1/1000 Cell Signalling 2217
Anti-Phospho-mTOR (Ser2448) (D9C2) Rabbit 1/1000 Cell Signalling 5536
Anti- mTOR (7C10) Rabbit 1/1000 Cell Signalling 2983
Anti-Phospho-PRAS40 (Thr246) (C77D7) Rabbit 1/1000 Cell Signalling 2997
Anti-PRAS40 (D23C7) Rabbit 1/1000 Cell Signalling 2691
Anti-TBK1/NAK Rabbit 1/1000 Cell Signalling 3013
Anti-Phospho-TBK1/NAK (Ser172) (D52C2) Rabbit 1/1000 Cell Signalling 5483
Anti-Phospho-PDK1 (Ser241) Rabbit 1/1000 Cell Signalling 3061
Anti-PDK1 (D37A7) Rabbit 1/1000 Cell Signalling 5662

Validation	All antibodies were independently validated by the vendors (Merk-Millipore, BD Biosciences, Bilogend, DSHB,Abcam, Cell Signaling , Santa-Cruz, websites and product pages).

Eukaryotic cell lines

Policy information about cell lines

Cell line source(s)	p.A53T patient-derived and control iPSC lines (Kouroupi et al PNAS 2017), iCell DopaNeurons 01279 , Catalog No C1028, and a heterozygous (HZ) A53T allelic variant isogenic to iCell DopaNeurons, PD SNCA A53T HZ 01279, Catalog No C1113 were commercially available (Fujifilm Cellular Dynamics International). SH-SY5Y cell line was generously provided by Dr. Leonidas Stefanis.
Authentication	None of the cell lines used were authenticated.
Mycoplasma contamination	All cell lines were negative for mycoplasma contamination.
Commonly misidentified lines (See ICLAC register)	N/A
